# Non‐familial intergenerational interventions and their impact on social and mental wellbeing of both younger and older people—A mapping review and evidence and gap map

**DOI:** 10.1002/cl2.1306

**Published:** 2023-02-16

**Authors:** Fiona Campbell, Rebecca Whear, Morwenna Rogers, Anthea Sutton, Ellie Robinson‐Carter, Jane Barlow, Richard Sharpe, Stuart Cohen, Louise Wolstenholme, Joanna Thompson‐Coon

**Affiliations:** ^1^ Evidence Synthesis Group Population Health Sciences Institute Newcastle University Newcastle UK; ^2^ NIHR CLAHRC South West Peninsula (PenCLAHRC) University of Exeter Medical School Exeter UK; ^3^ NIHR PenCLAHRC, Institute of Health Research University of Exeter Medical School Exeter UK; ^4^ Health Economics and Decision Science, ScHARR University of Sheffield Sheffield UK; ^5^ Sensory Trust Cornwall UK; ^6^ Department of Social Policy and Intervention University of Oxford Coventry UK; ^7^ Public Health, Cornwall Council University of Exeter Medical School St. Austell UK; ^8^ NHS Kernow Clinical Commissioning Group St. Austell UK; ^9^ 0‐19 Services, Sheffield Childrens NHS FT Sheffield UK; ^10^ NIHR ARC South West Peninsula (PenARC) University of Exeter Medical School Exeter UK

## Abstract

**Background:**

Opportunities for social connection between generations in the UK have diminished over the last few decades because of changes in the way that we live and work. The decline in communal spaces such as libraries, youth clubs and community centres mean that there are fewer opportunities to meet and mix socially with other generations outside our own families. Increased working hours, improved technology, changes in family patterns, relationship breakdowns within families and migration are also believed to be contributory factors to generation segregation. There are many potential economic, social and political impacts of generations living separate and parallel lives, for example, higher health and social care costs, an undermining of trust between generations reduced social capital, a reliance on the media to form understanding of others’ viewpoints and higher levels of anxiety and loneliness. Intergenerational programmes and activities can take many forms and are delivered in many settings.  Evidence suggests that intergenerational activity can have a positive impact on participants, for example, in reducing loneliness and exclusion for both older people and children and young people, improving mental health, increasing mutual understanding and addressing important issues such as ageism, housing and care. There are currently no other EGMs that exist that address this type of intervention; however, it would complement existing EGMs addressing child welfare.

**Objectives:**

To identify, appraise and bring together the evidence on the use of intergenerational practice, to answer the following specific research questions:

What is the volume, nature and diversity of research on, and evaluation of, intergenerational practice and learning?
What approaches have been used to deliver intergenerational activities and programmes that may be relevant to providing such services during and in the subsequent recovery from the COVID‐19 pandemic?
What promising intergenerational activities and programmes have been developed and are being used but have not yet been subject to formal evaluation?

**Search Methods:**

We searched MEDLINE (via OvidSp), EMBASE (via OvidSp), PsycINFO (via OvidSp), CINAHL (via EBSCOHost), Social Policy and Practice (via OvidSp), Health Management Information Consortium (via OvidSp), Ageline (via EBSCOhost), ASSIA (via ProQuest), Social Science Citations Index (via Web of Science), ERIC (via EBSCOhost), Community Care Inform Children, Research in Practice for Children, ChildData (via Social Policy and Practice), the Campbell Library, the Cochrane Database of Systematic Reviews and the CENTRAL database between 22 and 30 July 2021. We searched for additional grey literature via the Conference Proceedings Citation Index (via Web of Science) and ProQuest Dissertation & Theses Global and via relevant organisation websites, for example, Age UK, Age International, the Centre for Ageing Better, Barnado's, Children's Commission, UNICEF, Generations Working Together, the Intergenerational Foundation, Linking Generations and The Beth Johnson Foundation) and the Ottawa initiative called Older Adults and Students for Intergenerational support.

**Selection Criteria:**

Any intervention that brings older and younger people together with the purpose of interacting to achieve positive health and/or social and/or educational outcomes from any study design including systematic reviews, randomised controlled studies, observational studies, surveys and qualitative studies are included. The titles and abstracts, and later full texts, of records identified by the search methods were screened against inclusion criteria by two independent reviewers.

**Data Collection and Analysis:**

Data extraction was undertaken by one reviewer and checked by a second with any inconsistencies identified and resolved through discussion. The data extraction tool was developed on EPPI reviewer and was modified and tested through stakeholder and advisor consultation, and piloting of the process. The tool was informed by the research question and the structure of the map. We did not undertake quality appraisal of the included studies.

**Main Results:**

Our searches identified 12,056 references, after screening 500 research articles were included in the evidence gap map conducted across 27 countries. We identified 26 systematic reviews, 236 quantitative comparative studies (of which 38 were randomised controlled trials), 227 were qualitative studies (or had a qualitative element), 105 were observational studies (or had elements of observational methods) and 82 used a mixed methods approach. The outcomes reported in the research cover mental health (*n* = 73), physical health (*n* = 62), attainment and knowledge (*n* = 165), agency (*n* = 174), mental wellbeing (*n* = 224), loneliness and social isolation (*n* = 54), attitudes towards the other generation (*n* = 283), intergenerational interactions (*n* = 196), peer interactions (*n* = 30) and health promotion (*n* = 23) and including mutual outcomes such as the impact on community (*n* = 37) and perceptions on the sense of community (*n* = 43). Gaps in the evidence that were identified include: research that reports on mutual, societal and community outcomes of intergenerational interventions; more research on interventions classified as levels 1–4 and level 7 on the Intergenerational Engagement Scale, mental health, loneliness, social isolation, peer interactions, physical health and health promotion outcomes in children and young people; health promotion in older people; outcomes centred on care giver wellbeing, mental health and attitudes; economic outcomes; process outcomes and adverse or unexpected outcomes.

**Authors’ Conclusions:**

Whilst a substantional amount of research on intergenerational interventions has been identified in this EGM, as well as the gaps identified above, there is a need to explore promising interventions not yet formally evaluated. Research on this topic is gradually increasing, and systematic reviews will be important to determine how and why interventions are or are not beneficial. However, the primary research needs to build more cohesively so that the findings can be comparable and avoid research waste. The EGM presented here will nevertheless be a useful resource for decision‐makers allowing them to explore the evidence with regard to the different interventions that may be relevant to their population needs and the settings or resources available to them.

## 1 PLAIN LANGUAGE SUMMARY

### 1.1 Large evidence base for impact of intergenerational interventions involving young and old, but many gaps in research

There is a considerable body of research evidence on intergenerational interventions and their impact on older people and children and young people. However, there are still many research gaps, and primary research could benefit from more consistency in outcome reporting.

### 1.2 What is this evidence and gap map about?

Opportunities for social connection between generations in the UK have diminished over the last few decades because of changes in the way that we live and work. The Office for National Statistics Community Life Survey 2020‐2021 reports that 6% of adults in the UK said they often or always felt lonely. People aged 16 to 24 were significantly more likely to report feeling lonely often or always, which is 11% of that age group. Nine percent of people aged 65 years and over reported the same.

Evidence suggests that intergenerational activity can have a positive impact on participants, for example, in reducing loneliness and exclusion for both older people and children and young people, improving mental health, increasing mutual understanding, and addressing important issues such as ageism, housing and care.

However, knowing what to implement, how and for whom is complex due to the lack of evidence about their effectiveness, transferability of effects across settings and cost‐effectiveness. This evidence gap map (EGM) identifies the nature, volume and types of intergenerational interventions found in the research literature. It identifies areas for future research and evidence synthesis to help decision makers make more informed choices.

### 1.3 What is the aim of this evidence and gap map (EGM)?

The aim of this EGM is to identify all the existing research evidence on intergenerational interventions to improve understanding about intergenerational activities in terms of the health and social care outcomes of older people, younger people and children, and to inform future research.

### 1.4 What studies are included?

The EGM includes 500 research articles of any design on intergenerational interventions that do not include family members. The evidence comes from 27 countries.

We identified 26 systematic reviews, 236 quantitative comparative studies (of which 38 were randomised controlled trials), 227 qualitative studies (or had a qualitative element), 105 observational studies (or had elements of observational methods) and 82 with a mixed‐methods approach.

### 1.5 What are the main findings of this EGM?

The most commonly reported outcomes for children and young people were attitudes towards older people, knowledge and attainment, and intergenerational interactions.

For older people the most commonly reported outcomes were mental wellbeing, agency, attitudes towards younger people, and intergenerational interactions.

We identified several gaps in the research, including research on mutual, societal and community outcomes, young people's mental health, loneliness, social isolation, peer interactions, physical health and health promotion, outcomes centred on caregiver wellbeing, mental health and attitudes, and adverse or unexpected outcomes, including economic outcomes.

Interventions were most commonly delivered in schools, in the community or in care homes.

Interventions most commonly involved activities related to sharing perspectives of being an older or younger person/child, spending time together, helping with chores, helping more generally within a school environment, mentoring, art and crafts to engage the generations together, learning or sharing music and playing games.

### 1.6 What do the findings of the map mean?

The EGM provides a starting point for researchers and decision makers to access the available research evidence on the effectiveness of intergenerational interventions.

The map demonstrates considerable diversity in the types of intergenerational activity. It also shows that it is mainly demonstration projects that are evaluated.

The quality of the evaluations makes analysis of their effectiveness, and hence their impact on shaping practice and policy, limited.

Methods of supporting useful evaluations of these types of interventions – so they are measuring meaningful outcomes – is needed. This EGM identifies many areas where there are still gaps in research.

### 1.7 How up‐to‐date is this EGM?

The authors searched for studies published up to July 2021.

## 2 BACKGROUND

### 2.1 Introduction

#### 2.1.1 The problem, condition or issue

Opportunities for social connection between generations in the UK have diminished over the last few decades because of changes in the way that we live and work (Kingman, 2016; United for all Ages, 2017). Housing and economic trends have seen younger people move to live in city centres whilst the older generation live in towns and rural areas. A report published by the Intergenerational Foundation in 2016 (Kingman, 2016) suggests that in the 25 biggest cities within the UK only 5% of people aged over 65 live in the same neighbourhood as someone under the age of 18. Furthermore, even when people from different age groups do live in the same area, the decline in spaces such as libraries, youth clubs and community centres mean that there are fewer opportunities to meet and mix socially with other generations outside our own families. Increased working hours, improved technology, changes in family patterns, relationship breakdowns within families and migration are also believed to be contributory factors to generation segregation (Generations Working Together, 2019). There are many potential economic, social and political impacts of generations living separate and parallel lives, for example, higher health and social care costs, an undermining of trust between generations (Brown & Henkin, 2014; R. L. Jones, 2011; Laurence, 2016; Vitman et al., 2013); reduced social capital (Laurence, 2016); a reliance on the media to form understanding of others’ viewpoints (Edström, 2018; Vasil & Wass, 1993) and higher levels of anxiety and loneliness. A review of the prevalence of loneliness in 113 countries found high levels of loneliness for a substantial proportion of the population in many countries (Surkalim et al., 2022). For example, in the Office for National Statistics Community Life Survey, 2020 to 2021 (ONS, 2021); 6% of adults in the UK reported feeling lonely often or always. Those aged 16–24 were also significantly more likely to report feeling lonely often or always (11% of that age group) with 9% of those aged 65 years and over report the same.

#### 2.1.2 The intervention

Intergenerational programmes and activities can take many forms and are delivered in many settings, very often by third sector organisations. Although evidence suggests that intergenerational activity can have a positive impact on participants (e.g., reducing loneliness and exclusion for both older people and children and young people, improving mental health, increasing mutual understanding and addressing important issues such as ageism, housing and care), commissioning decisions are complex due to the apparent wealth of options available, and yet limited and varying resources with which to provide them. This evidence gap map brings together all the available research evidence on intergenerational interventions.

### 2.2 Why it is important to develop the EGM

Intergenerational programmes and activities are promising interventions that can address some of the needs of both children and young people and older people. The outcomes for children and young people and older people will form one of the key dimensions for the EGM—the list of which were developed from the frameworks listed below and through discussion with our stakeholder advisory group. The other dimension will be type of intergenerational intervention as categorised by the Depth of Intergenerational Engagement Scale (Kaplan, 2004). These two dimensions will give an overall picture of broad types of interventions and outcomes that have, and have not, been researched. Intergenerational interventions can take many forms and are delivered in diverse settings, therefore it will be important to be able to distinguish which aspects and characteristics of the interventions are supported by the evidence. We will therefore use the filter function in the EGM to identify the research design, intervention setting, age of the children/young people involved, the focus or activities involved, and any participant characteristics that have been targeted by an intervention.

Although evidence suggests that intergenerational activity can have a positive impact on participants, commissioning decisions are complex due to the lack of evidence about their effectiveness, transferability of effects across settings, and cost‐effectiveness. This evidence and gap map (EGM) will identify the nature, volume and types of intergenerational interventions that have been undertaken and evaluated. It will identify areas for future research and evidence synthesis.

There are currently no other EGMs that exist that address this type of intervention; however, it would complement existing EGMs addressing child welfare.

## 3 OBJECTIVES

We aim to use existing evidence to improve understanding about intergenerational activities in terms of the health and social care outcomes of older people, younger people and children.

Our objectives are to:

Identify and bring together the evidence on the use of intergenerational practice, to answer the following specific research questions:

- What is the volume, nature and diversity of research on, and evaluation of, intergenerational practice and learning?
- What approaches have been used to deliver intergenerational activities and programmes that may be relevant to providing such services during and in the subsequent recovery from the COVID‐19 pandemic?
- What promising intergenerational activities and programmes have been developed and are being used but have not yet been subject to formal evaluation?

## 4 METHODS

### 4.1 EGM: Definition and purpose

EGMs are maps of a specific sector or subsector which typically includes both systematic reviews and primary studies. Produced using the same systematic approach as systematic reviews, EGMs usually show what evidence is there, not what the evidence says (White et al., 2018).

The EGM framework will inform the inclusion and exclusion criteria of the EGM. Here, we describe the population, intervention, comparison, outcomes (indicators) and study designs for the map.

### 4.2 Framework development and scope

The aim of this EGM is to capture the broad range of evidence from systematic reviews and primary research that has investigated intergenerational practice.

The EGM will enable policymakers and practitioners in the field to take account of the research evidence in the commissioning and use of intergenerational practice in health and social care. It will also highlight opportunities for intergenerational activities and programmes during and in the subsequent recovery from the COVID‐19 pandemic and direct the commissioning of appropriate research where there are evidence gaps.

The scope of the EGM is defined by a framework of interventions and outcomes presented as two dimensions: the rows include interventions with sub‐categories, and the columns outcome domains. The framework was developed in consultation with our stakeholders who identified how the interventions could be helpfully defined using an existing framework which categorises interventions based on the level of engagement they promote Depth of Intergenerational Engagement Scale (Kaplan, 2004). We identified several outcomes that the research literature in this area already reports on, however we were aware that using the literature alone does not help us to identify outcomes that may be of interest but are not reported on. To address this issue, we asked our stakeholders to review the list of outcomes we had drawn from the literature and suggest additional outcomes that they felt were also of interest/importance. All these outcomes were then captured in the framework for the map. For the benefit/ease of those using the map the outcomes were grouped into the following subsections, outcomes for children and young people, outcomes for older people, mutual outcomes, for example, community, outcomes for others, for example, carers, economic outcomes, process outcomes and adverse or unexpected outcomes, so that they could be expanded or collapsed depending on the preferences of the user.

Further attributes can be considered and used to filter the results, such as the research design of the included studies or characteristics of the included populations, for example, age of the younger people, any people with vulnerable or protected characteristics. Each cell shows studies which contain evidence on that combination of intervention and outcome. Study characteristics including, for example, study design, setting, intervention level and intervention activity/focus are coded, and the evidence can be filtered by these characteristics.

### 4.3 Stakeholder engagement

The following individuals have contributed to the project through the advisory group:

Ronald Amanze; Iain Lang—University of Exeter; Vicki Goodwin—University of Exeter; Jo Day—University of Exeter; Aideen Young ‐ Centre for Ageing Better; G.J. Melendez Torres—University of Exeter; Dylan Kneale—UCL; Ruth Garside—University of Exeter; Claire Goodman—University of Hertfordshire; Tracey Howe—Cochrane Campbell Global Ageing Partnership; Kelvin Yates—AgeUK Cornwall; Nathan Hughes—University of Sheffield; Debbie Hanson—Sheffield City Council; Laura Abbott—Chilypep; Hannah Fairbrother—University of Sheffield; Kerry Albright—Unicef; Rachel Staniforth—Public Health; Girish Vaidya—Sheffield Children's NHS Foundation Trust; Sally Pearse—Sheffield University.

Members of the ‘Only Connect!’ Network have contributed throughout the project. The group includes local, national and international members from the care sector, local government, academia, people living with dementia, schools and leading organisations involved in providing intergenerational activities. Members of the group also facilitated discussion of the project with older people, people living with dementia, and young people with experience of taking part in intergenerational activities.

We convened three virtual whole project meetings to include stakeholders and advisory group members (during Months 1 and 3), which assisted with understanding and presentation of the evidence in the EGM. We used break‐out rooms and other methods of sharing ideas and suggestions such as a JamBoard and individual meetings to ensure that as many views and perspectives were captured as possible. We followed large meetings up with smaller meetings/phone calls where necessary.

Between meetings we involved people through email, telephone and video conferencing, depending on the nature of the involvement and the preference of individuals.

During the stakeholder meeting in month one the stakeholder group informed the development of the framework, which helped to form the matrix for the EGM. Working in smaller groups, we encouraged participants to identify outcomes and types of intervention. This was used, along with the wider literature to inform the components of the framework.

### 4.4 Conceptual framework

**Figure 1:**
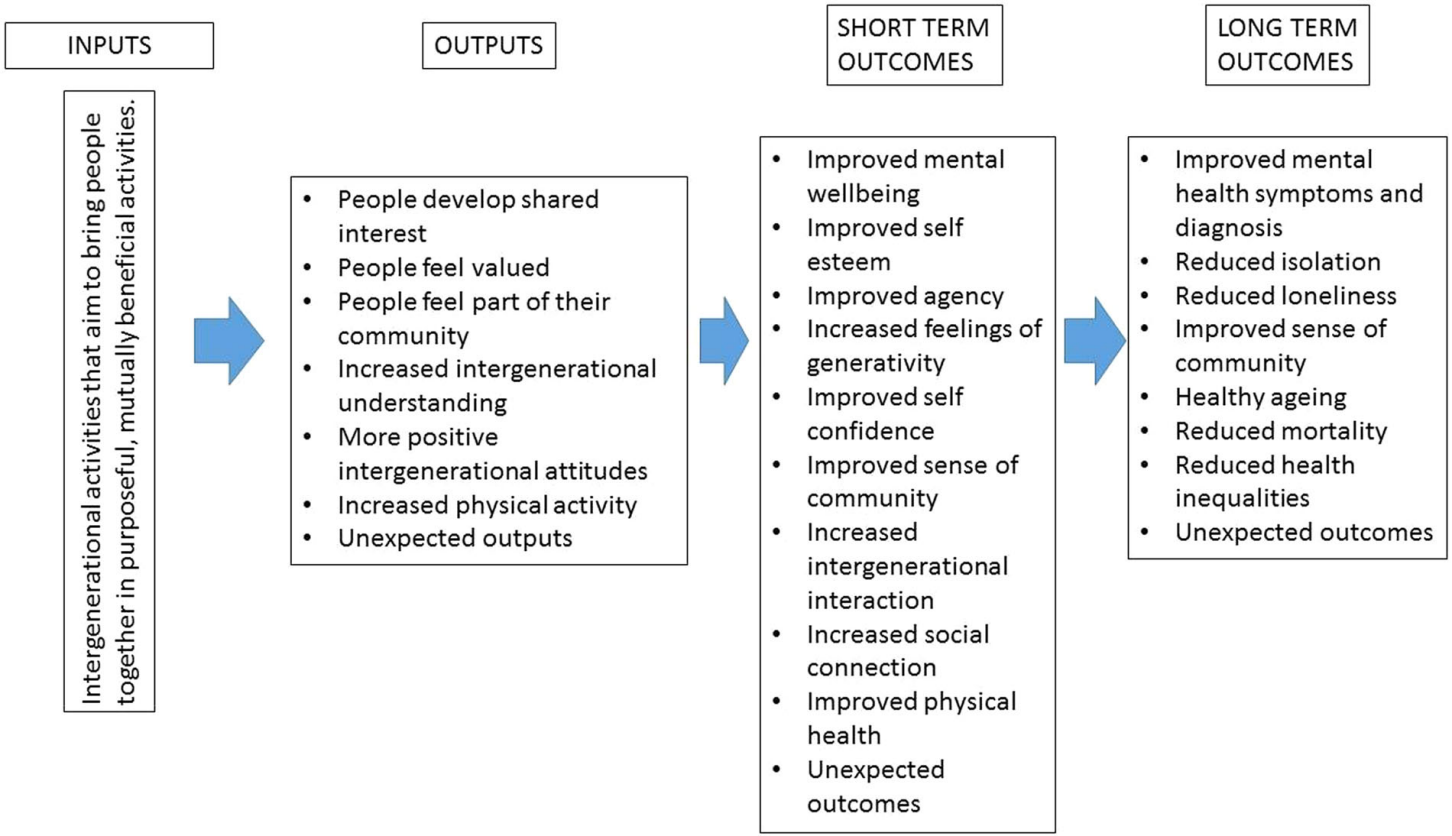
Logic model.

We developed a broad logic model to portray the general theory/pathway expected in any intergenerational intervention (Figure 1).

Our conceptual framework is informed by the following: the five essential elements of wellbeing described by Nazroo and colleagues adopted by the Institute for Public Policy Research (IPPR) (Nazroo et al., 2005); the seven outcomes outlined in the Department of Health Social Care Green Paper, Independence, Well‐being and Choice (DOH, 2005) and the six domains identified in which actions are required for child and adolescent health and wellbeing by the World Health Organisation and UNICEF (UNICEF & WHO, 2020).

These were then further considered and discussed with our stakeholders to identify the relevant outcomes of interest for one dimension of the framework. The other dimension of the framework was informed by the Depth of Intergenerational Engagement Scale (Kaplan, 2004) which gives a broad category on intervention based on the level of engagement it requires between the two generations. As intergenerational interventions are delivered using different formats and in diverse settings, it is important to be able to easily identify intervention characteristics such as research design, intervention setting, age of the children/young people involved, the focus or activities involved and any participant characteristics that have been targeted by an intervention. We will use the filter function in the EGM to capture and present these and these are further detailed below. We expect the interventions to cover both universal and targeted approaches, and whilst these definitions are not explicitly used as a filter in the map, targeted approaches will be identifiable by the filters used to describe particular characteristics of the populations involved in the intervention.

**Table:** 

Five essential elements of wellbeing (Nazroo et al., 2005)	Seven outcomes in the social care Green Paper, Independence, Well‐being and Choice (DOH, 2005)	Six domains identified in which actions are required for child and adolescent health and wellbeing (UNICEF & WHO, 2020)
Resilience	Improved health and emotional well‐being	Good health
Independence	Improved quality of life	Adequate nutrition
Health	Making a positive contribution	Opportunities for learning and education
Income and wealth	Increased choice and control	Securing, safety and a supportive clean environment
Having a role and having time	Freedom from discrimination or harassment	Responsive relationships and connectedness
	Economic well‐being	Realisation of personal autonomy and resilience
	Maintaining personal dignity and respect	

### 4.5 Dimensions

#### 4.5.1 Types of study design

We wanted to capture all the available evidence (not just intervention effectiveness) regarding intergenerational interventions for users to be able to use the EGM to identify any research they were interested in and where the gaps in evidence still lie. Therefore, all study designs including systematic reviews, randomised controlled studies, observational studies, surveys and qualitative studies are included. Due to the substantial amount of research literature found we did not include news items describing intergenerational activities and programmes even if they reported innovative interventions not otherwise represented within the evidence base (as in the protocol).

#### 4.5.2 Types of intervention/problem

We included any intervention that brings older and younger people together with the purpose of interacting to achieve positive health and/or social and/or educational outcomes. These include reminiscence programmes, buddy systems, storytelling, school‐based interventions and arts‐based interventions as well as others. We used the Depth of Intergenerational Engagement Scale (Kaplan, 2004) as the framework for the interventions. This is described below:

##### 4.5.2.1 The Depth of Intergenerational Engagement Scale

The Depth of Intergenerational Engagement Scale places programmes and activities on a continuum, with points that correspond to different levels of intergenerational engagement, ranging from initiatives that provide no direct contact between age groups (point 1) to those that promote intensive contact and ongoing opportunities for intimacy (point 7). Examples of intergenerational initiatives fitting into each point on the scale are described.

1. Learning about other age groups
  Participants learn about the lives of persons in other age groups, although there is no direct or indirect contact.
  Example: ‘Learning about Aging’ programmes designed to teach youth about aspect(s) of the aging process.
2. Seeing the other age group at a distance
  These initiatives facilitate an indirect exchange between individuals of two or more age groups. Participants might exchange videos, write letters, or share artwork with each other, but never actually meet in person.
  Example: A pen‐pal programme in which youth in an after‐school club exchange letters with residents of a nursing home.
3. Meeting each other
  Initiatives culminate in a meeting between the young participants and older adults, generally planned as a one‐time experience.
  Example: A class of students plan for and visit a local senior centre in which all engage in activities during a July 4th picnic.
4. Annual or periodic activities
  Often tied to established community events or organisational celebrations, intergenerational activities occur on a regular basis. Although infrequent, these activities might symbolise intergenerational and community unity and influence attitudes and openness towards additional or ongoing activities.
  Examples: Intergenerational activities at a school on Grandparent's Day, an annual community dance in which youth and older adults are actively involved, and Christmas caroling at assisted‐living homes.
5. Demonstration projects
  Demonstration projects generally involve ongoing intergenerational activities over a defined period of time. Depending on project goals and objectives, the intergenerational exchange and learning can be quite intensive. These initiatives are often implemented on an experimental or trial basis, and frequently depend on external funding.
  Example: A 6‐month pilot programme, sponsored by an agency that provides teen parenthood support services. Senior adults who have successfully raised children are enlisted to mentor and provide support for pregnant and parenting teens.
6. Ongoing intergenerational programmes
  Programmes from the previous category that have been deemed successful and valuable from the perspective of the participating organisations and the clientele are incorporated as an integral part of their operation. This extends to programme and staff development such as preparing individuals to work with populations of various age groups.
  Example: Based on a partnership forged between a senior centre, a community youth centre, and an environmental education centre, senior adults and youth plan and execute the town's environmental improvement campaign. Systems are established to organise numerous projects, train and assign participants, and provide continuing support and recognition.
7. Ongoing, natural intergenerational sharing, support and communication

There are times when the intergenerational reconnection theme transcends a distinct programme or intervention. This is evident when the social norms, institutional policies and priorities of a particular site, community, or society reflect values of intergenerational reciprocity and interdependence. Intergenerational engagement takes place as a function of the way community settings are planned and established. In this context, opportunities for meaningful intergenerational engagement are abundant and embedded in local tradition.

Example: A YMCA facility houses a senior citizen centre. Older adults and youth participate in a variety of age‐integrated activities.

Programmes fitting into all points on this continuum provide positive experiences for interacting with persons in other age groups. However, if the aim is ambitious, such as changing attitudes about other age groups, building a sense of community, enhancing self‐esteem, or establishing nurturing intimate relationships, it becomes important to focus on programmes that fit into Levels 4–7 on the scale. Programmes would take place over an extended period of time, would last anywhere from a few months to many years, and would provide extensive interaction opportunities (Kaplan, 2004).

#### 4.5.3 Types of population

Older adults and children and young people. No age boundary restrictions were applied but we sought studies that suggest at least one skipped generation between the older and younger participants. Studies in which participants were related by family or marriage were excluded. Inclusion was not determined by prior age cut‐offs but by the included studies own definition of ‘older people’ and ‘young people’.

#### 4.5.4 Types of outcome measures

We included all reported outcomes. Outcomes did not form part of the criteria for including studies in the EGM since we are keen to explore all of the available evidence.

#### 4.5.5 Other eligibility criteria

##### Types of settings

Any setting or context. No restrictions on language.

##### Status of studies

We included studies irrespective of their publication status and their electronic availability. We also aimed to include ongoing studies where it was feasible to ascertain when the study will be completed.

### 4.6 Search methods and sources

We searched MEDLINE (via OvidSp), EMBASE (via OvidSp), PsycINFO (via OvidSp), CINAHL (via EBSCOHost), Social Policy and Practice (via OvidSp), Health Management Information Consortium (via OvidSp), Ageline (via EBSCOhost), ASSIA (via ProQuest), Social Science Citations Index (via Web of Science), ERIC (via EBSCOhost), Community Care Inform Children, Research in Practice for Children, ChildData (via Social Policy and Practice), the Campbell Library, the Cochrane Database of Systematic Reviews and the CENTRAL database between 22nd and 30th July 2021.

We used terms covering intergenerational practice, or terms for older adults combined with terms for children and intergenerational activities. The full search strategies for every database are available in Supporting Information: Appendix 1. We searched for additional grey literature via the Conference Proceedings Citation Index (via Web of Science) and ProQuest Dissertation & Theses Global.

We expected that some relevant reports would not be published in academic sources so we also searched for grey literature via relevant organisation websites, for example, Age UK, Age International, the Centre for Ageing Better, Barnado's, Children's Commission, UNICEF, Generations Working Together, the Intergenerational Foundation, Linking Generations and The Beth Johnson Foundation and the Ottawa initiative called Older Adults and Students for Intergenerational support (OASIS, https://www.oasis-aesi.com/) between 28 January 2022 and 4 February 2022 by either examining the resources section of the website or entering ‘intergenerational’ into the search box.

Due to the amount of research literature found we limited our additional searches (forwards and backwards citation chasing) as follows: we carried out backward citation chasing on the included systematic reviews to identify any randomised controlled trials (RCTs) and other systematic reviews not already included in the EGM; we did not check the citations of older key papers (forward citation chasing); we hand searched one key—journal the Journal of Intergenerational Relationships. Although we did not conduct the horizon scanning process described in the protocol we expect to conduct that in subsequent reviews.

We published the agreed protocol with Campbell (Thompson‐Coon et al., 2022).

### 4.7 Analysis and presentation

#### 4.7.1 Report structure

The report provides tabulations or graphs of the number of studies, with accompanying narrative description, by

- Intervention category and subcategory
- Outcome domain and subdomain
- Table of ‘aggregate map’ of interventions and outcomes
- Country (designated by country of first author)
- Year
- Study type
- Population subgroups.

The interactive EGM can also be used to explore the data using the filters presented below.

#### 4.7.2 Filters for presentation

In addition to the interventions and outcomes, the following filters have been coded:

Characteristics of the participants that the intervention targets (this was an iterative list that also aimed to include characteristics included in Progress Plus (O'Neill et al., 2014)

Progress plus:

- Minority groups (in either generation based on race, ethnicity, culture, language, LGBTQ)
- Low socioeconomic status (in either generation)
- Unemployment (in either generation)
- Educational needs (in either generation
- Social isolation (in either generation)

Other important characteristics (discussed with the Stakeholder advisory group):

- Mental health difficulties (*in either generation*)
- Physical health difficulties (*in either generation*)
- Age category of the *children/young people*—0–5 years, 6–12 years, 12–18 years, 19–30 years.
- *Children* experiencing childhood adversity
- *Older peopl*e with cognitive impairment

Contextual factors:

- Country/region—country of the first author
- Setting—where the intervention took place, for example, in school, care home, retirement village, university/higher education, shared facility, day care centre, hospital, assisted living centre or community setting

Study design factors:

- Study design—RCTs, non‐RCTs, interrupted time series, controlled before and after studies, observational studies, qualitative studies, mixed methods and systematic reviews

Focus of the intervention (the activities involved in the intervention):

- Education—where older or younger generations teach the other generation a skill or share educational knowledge
- Art—generations share in arts or crafts
- Music—generations share musical activities or teach a musical skill
- Interaction—interaction between the generations like conversation, spending time/communication, helping tasks
- Cooking—generations cooking together
- Dance—generations sharing and working together in dance performances
- Drama—generations sharing and working together in dramatic performances
- Environmental activities—generations sharing environmental activities
- Exercise—generations exercising together or helping the other generation to exercise more
- Gardening—generations gardening together
- History—older generations helping to share history with younger generations
- IT—younger generations helping older generations to learn and use technology
- Language—older generations helping younger generations to learn/practice language
- Letter writing—generations writing to each other and to help learn to write
- Literature—generations sharing literature together
- Living together—generations living in the same space (usually students/young adults living with older generation—with no familial connection)
- Maths—older generation helping younger generation to learn Maths
- Playing games—generations playing games together
- Professional education—older generation involved in professional education of students working with older generations
- Reading—older generation helping younger generation to learn to read
- Reminiscence—older generations encouraged to reminisce by presence of younger generation
- Science activities—generations conduct science activities together
- Sharing meals—generations share a meal together
- Sharing perspectives (of being and older person/a child/young person)
- Story telling—one generation tells a story to another
- Trips and excursions—generations visit places or attend events together
- Other—any intervention not covered by the descriptions above, for example, general presence/assistance in a school context.

#### 4.7.3 Dependency

Each entry in the map is a systematic review or a primary study of effectiveness. The final EGM identifies the number of studies covered by the map in each sector or subsector. We have included all relevant systematic reviews and primary studies irrespective of whether there is overlap between reviews and studies. Similarly, studies with multiple interventions or multiple outcomes may appear multiple times within the map.

### 4.8 Data collection and analysis

#### 4.8.1 Screening and study selection

The titles and abstracts of records identified by bibliographic and supplementary search methods were screened against inclusion criteria by two independent reviewers (FC, JTC, RW, MR) looking for reasons for exclusion. The full text of records retained at this stage were retrieved and screened for inclusion against the inclusion criteria using the same process. All included studies were saved in a master library using EndNote X8 Endnote X8. These studies were then entered on to EPPI reviewer where the remaining data extraction and management was conducted. These are the studies that form the basis for the EGM and that can also be used in the next phase of this project, for example, subsequent review topics.

#### 4.8.2 Data extraction and management

Data extraction was undertaken by one reviewer and checked by a second (FC, JTC, RW, MR) with any inconsistencies identified and resolved through discussion. The data extraction tool was modified and tested through stakeholder and advisor consultation, and piloting of the process. The tool was informed by the research question and the structure of the map. Data extraction was conducted using EPPI reviewer (Thomas et al., 2022).

We extracted data on study design, geographical location, setting, population (age, gender, health condition/status, equity characteristics), intervention (type, mode of delivery, setting) and outcomes.

We used the PROGRESS‐Plus framework (O'Neill et al., 2014) to identify studies that measured effects of interventions by gender or other factors that may lead to health inequalities (e.g., ethnicity; etc.).

#### 4.8.3 Tools for assessing risk of bias/study quality of included reviews

We did not undertake quality appraisal of the included studies.

#### 4.8.4 Methods for mapping

We used EPPI‐Reviewer software (Thomas et al., 2022) for data extraction and coding, and to generate the online EGM (EPPI Mapper 2022). The map is interactive so that users can click on (i) cells within the matrix to show a list of the relevant studies and on (ii) study names to access the study or a reference and database link for the study.

## 5 RESULTS

### 5.1 Description of studies

#### 5.1.1 Results of the search

**Figure 2:**
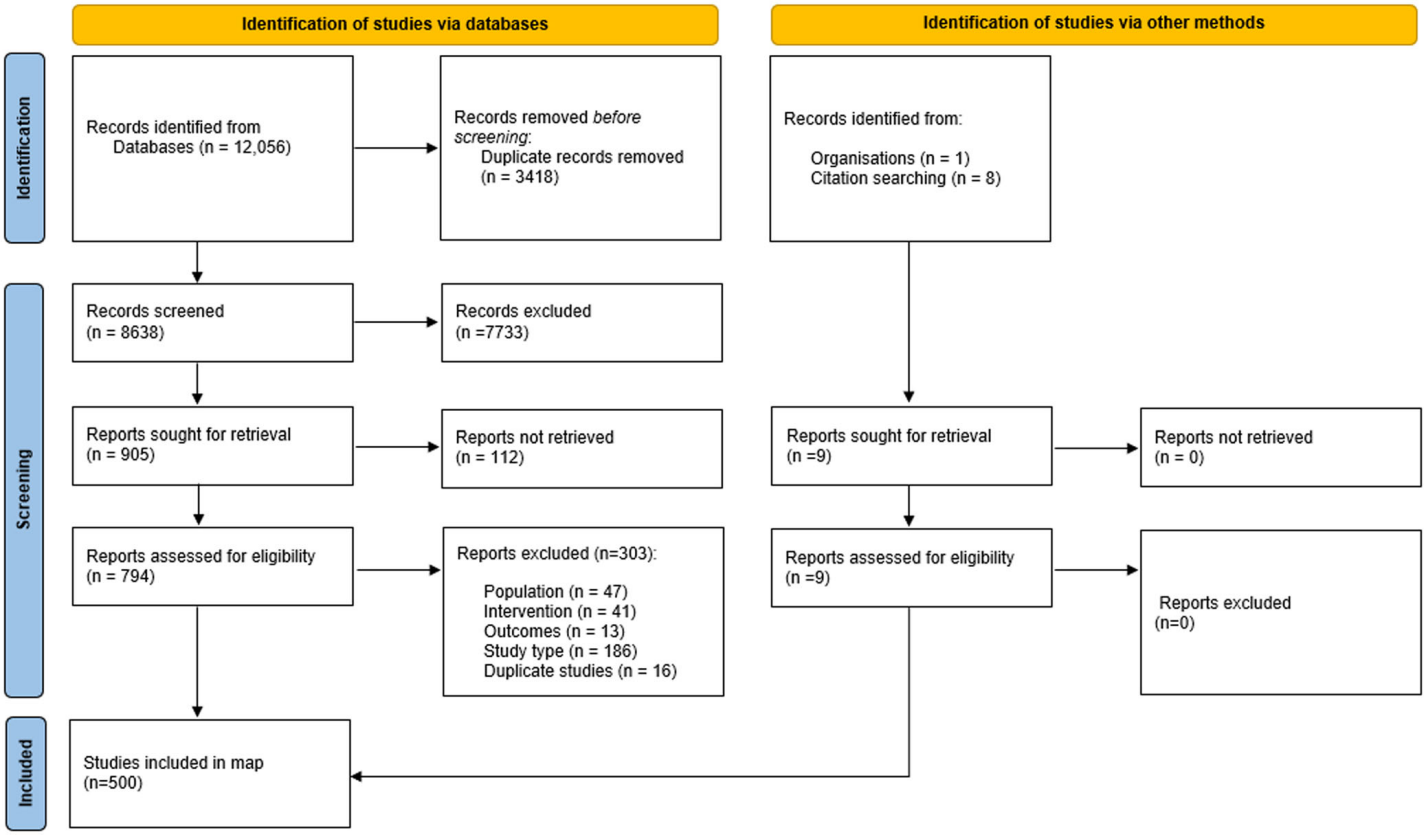
Prisma flow diagram.

Our search strategy identified 12,056 references (reduced to 8638 after removal of duplicate studies). After both stages of screening had been completed a total of 500 research articles were included in the EGM. Figure 2—PRISMA flow diagram provides further details on the screening process and decisions at each stage (Page et al., 2021).

Studies were conducted in 27 countries (based on country of first author). Studies were conducted in the US (*n* = 326), Canada (*n* = 33), the UK (*n* = 29), Australia (*n* = 27), Japan (*n* = 15), Spain (*n* = 8), Hong Kong (*n* = 7), Italy (*n* = 7), South Korea (*n* = 5), Brazil, France, Portugal, Singapore, Taiwan, Israel (*n* = 4 in each), Sweden, the Netherlands (*n* = 3 in each), Germany, Ireland, China (*n* = 2 in each), one each in Austria, Finland, Greece, Malta, New Zealand, South Africa and Switzerland.

The 500 research studies were published over a period of 46 years from 1975 to 2021. All study designs were included, we identified 26 systematic reviews, 236 quantitative comparative studies (of which 38 were RCTs), 227 were qualitative studies (or had a qualitative element), 105 were observational studies (or had elements of observational methods) and 82 used a mixed methods approach. We did not record the age of the older generations involved in the intergenerational interventions as we were looking more closely for evidence of a generational gap between the two populations; however, we did record the ages of the young people and children involved in the interventions which spanned from 0 to 30 years. One hundred and twenty‐two interventions involved children aged between 0 and 5 years, 182 interventions involved children aged 6–12 years, 137 interventions involved young people aged 12–18 years, and 155 interventions involved young people aged 19–30 years. In 39 intervention studies the age range could not be established.

**Figure 3:**
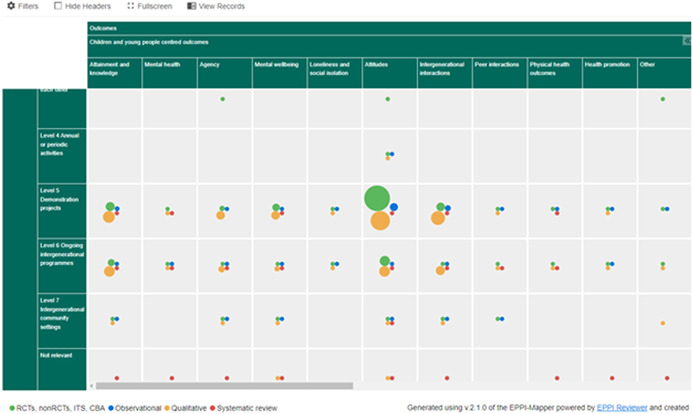
Figure 3 EGM aggregate map interventions × outcomes (children and young people).

**Figure 4:**
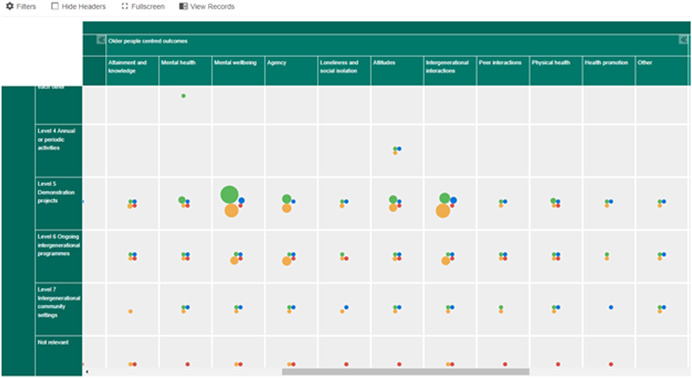
Figure 4 EGM aggregate map interventions × outcomes (older people).

**Figure 5:**
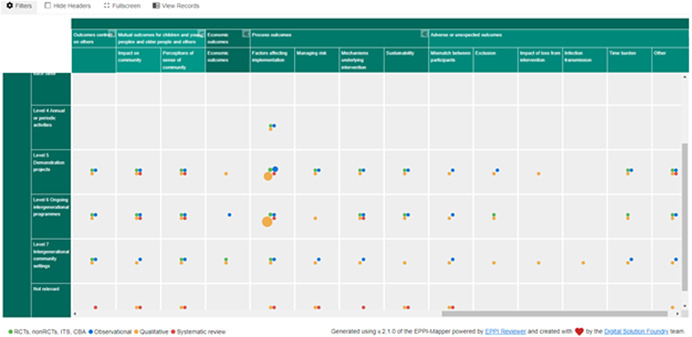
Figure 5 EGM aggregate map interventions × outcomes (other people, mutual outcomes, economic outcomes, process outcomes, adverse or unexpected outcomes).

Outcomes included (but were not limited to) social isolation, engagement, interacting, perception of people living with dementia, social inclusion, psychological outcomes, depression, anxiety, social skills, self‐confidence, creativity, school performance, relationship building, attitudes, empathy, personal growth, community responsibility, activity levels (physical activities), mood, quality of life, stimulation of memory and mind, digital inclusion (helping people to get online). Figures 2, 3, 4 depict snapshots of how the EGM looks and how the studies are presented across the dimensions of intervention level and outcomes for children and young people (Figure 3), older people (Figure 4) and outcomes other people (e.g., carers), mutual outcomes (e.g., sense of community), economic outcomes, process outcomes, and adverse or unexpected outcomes (Figure 5).

#### 5.1.2 Excluded studies

Of the 794 reports assessed for eligibility, 303 reports were excluded. One‐hundred and eighty‐six reports were excluded as they were considered the wrong study type, for example, reports that did not detail their research methods, descriptions or summaries of interventions, or were personal reports/descriptions of an intervention; 47 were excluded because they included the wrong population, for example, where ‘intergenerational’ referred to a spread across generations or where a generational gap could not be ascertained; 41 were excluded based on ineligibility of the intervention, for example, where the generations did not have direct contact or interact; 13 were excluded because they did not report on participant outcomes; 16 were excluded as they were duplicate reports. All 303 excluded studies are listed in Supporting Information: Appendix 2.

#### 5.1.3 Studies awaiting classification (if applicable)

None identified.

### 5.2 Synthesis of included studies

The interactive map can be found here.

#### 5.2.1 Intervention level

We used the Depth of Intergenerational Engagement Scale (Kaplan, 2004) as the framework for describing the interventions identified in this EGM. By the nature of the eligibility criteria for this EGM interventions that would have been classified as Level 1 (Learning about other age groups—participants learn about the lives of persons in other age groups, although there is *no direct or indirect contact*) or level 2 (Seeing the other age group at a distance—these initiatives facilitate an *indirect* exchange between individuals of two or more age groups. Participants might exchange videos, write letters, or share artwork with each other, but never actually meet in person) are not represented as they did not meet the eligibility criterion with regard to the generations having direct contact/interaction with each other.

In Table 1 we can see that the included interventions most commonly fall within Level 5 (Demonstration projects—generally involve ongoing intergenerational activities over a defined period of time, *n* = 284) or Level 6 (Ongoing intergenerational programmes—Programmes from the previous category that have been deemed successful and valuable from the perspective of the participating organisations, *n* = 155) with a seemingly increasing (based on the frequency of published studies in the last 5 years) number of Level 7 interventions (Ongoing, natural intergenerational sharing, support and communication—evident when the social norms, institutional policies and priorities of a particular site, community, or society reflect values of intergenerational reciprocity and interdependence, *n* = 35). This is what we would expect to see when looking for *research* in this area because interaction between generations described in interventions in Levels 3 and 4 is less likely to conform to an intervention that could be tested in a research study. However, this doesn't mean that this type of interaction is not being facilitated by organisations in practice.

**Table 1:** Study design of evidence present in each intervention level.

Intervention level/study design	Systematic review	RCT	Non‐RCT	Qualitative	Observational	Mixed methods	Total studies in EGM *
1	0	0	0	0	0	0	0
2	0	0	0	0	0	0	0
3	0	1	3	0	0	0	4
4	0	0	1	1	0	1	1
5	0	23	131	122	63	52	284
6	0	14	53	82	28	25	155
7	0	0	11	19	14	4	35
Total	26	38	198	227	105	82	

*Note*: *This is the number of studies at this level in the EGM some studies are represented in more than one study design category hence this number does not represent the total number in the relevant row.

Abbreviation: RCT, randomised controlled trial.

Some examples of the interventions identified in Levels 3–7 are:

Level 3—Developing one‐one relationships via instagram (Lytle et al., 2020) or the Intergenerational Partners Project where 4th Grade students share activities with older people to develop friendships (Aday et al., 1996).

Level 4—An intergenerational dinner event where medical students and older people attended together and participated in dancing and games together (Diachun et al., 2007; Dumbrell et al., 2007).

Level 5—These are demonstration projects aiming to see if an intervention can become a more permanent/sustainable intergenerational activity including, but not exclusively, projects that might target specific populations. For example, an intervention aiming to increase the citizenship experience of young children and their awareness of what it means to live with stroke tackling social isolation and self‐confidence in older people with stroke whilst encouraging mutual fine motor skill development such as handwriting (Lane, 2016).

Level 6—Ongoing interventions that are relatively well established, such as service learning opportunities for students studying topics where intergenerational interactions will aid their learning and development of personal skills related to future employment (Howell et al., 2021); for example or the ‘Through their Eyes Project’ where health sciences students are partnered with older adults to explore and assess the age‐friendliness of their neighbourhood (Gardner & Alegre, 2019); or ‘Active Generations’ an intergenerational nutrition education and activity programme implemented in out‐of‐school environments (after school and summer camps) where older adult volunteers implement a version of the evidence‐based childhood obesity prevention programme, ‘Coordinated Approach to Child Health’ (Werner et al., 2012).

Level 7—Where younger generations might live with older generations in intergenerational housing projects (Hock & Mickus, 2019; Kilaberia & Ratner, 2018; Labit & Dubost, 2016) or where very young children (0–5years) have their nursery/kindergarten located within a care home setting (Doll & Bolender, 2010; Rosa Hernandez et al., 2020; Skropeta et al., 2014).

#### 5.2.2 Outcomes reported

Table 2 summarises broad categories of outcomes reported across the included studies and also shows how these varied depending on the age of the young people or children involved in the study. Interestingly we found that not all research in this area reported on the outcomes for both generations; some intervention studies only reported on outcomes or experiences for one of the generations with the opposite generation being considered part of the intervention itself.

**Table 2:** Broad outcomes reported across included studies.

Age group/outcome	Children/younger people's outcomes	Older people's outcomes	Other people's outcomes (e.g., carers)	Mutual outcomes	Economic outcomes	Process outcomes	Adverse outcomes
0–5	71	94	17	16	3	47	19
6–12	137	115	18	26	1	65	15
12–18	103	99	12	26	0	55	9
19–30	129	89	10	12	0	56	11
Not described	25	35	1	10	0	20	8

More specifically the outcomes reported in the research identified in this EGM cover mental health (*n* = 73), physical health (*n* = 62), attainment and knowledge (*n* = 165), agency (*n* = 174), mental wellbeing (*n* = 224), loneliness and social isolation (*n* = 54), attitudes towards the other generation (*n* = 283), intergenerational interactions (*n* = 196), peer interactions (*n* = 30) and health promotion (*n* = 23) and including mutual outcomes such as the impact on community (*n* = 37) and perceptions on the sense of community (*n* = 43). The most commonly reported children/younger people's outcomes were attitudes towards older people, knowledge and attainment and intergenerational interactions. For older people the most commonly reported outcomes were mental wellbeing, agency, attitudes towards younger people and intergenerational interactions.

Economic outcomes (*n* = 3) and adverse or unexpected outcomes (*n* = 47) were not commonly reported but process outcomes such as factors affecting implementation, and mechanisms of interventions were reported across 183 studies.

Of those reporting adverse or unexpected outcomes (mostly from studies that used qualitative methods), 14 report time being a burden associated with the running of the intervention, 12 report a mismatch between the pairing of participants across the generations, which negatively impacted on the effects of the intervention, eight reported that some participants (or those around them) still felt excluded, three were concerned with the impact that loss might have on participants (particularly the loss of an older person with whom a younger person was interacting) and one study reported concerns about the risk around transmitting infections between older and younger participants. Other unexpected or adverse outcomes were also reported across 25 studies including negative behaviours and attitudes during interactions, and careful requirements for the design and implementation of interventions to ensure positive experiences and interactions.

Of the 183 studies reporting on process outcomes, 155 reported on factors affecting the implementation of the intervention being studied. The factors reported are dependent on the type of intervention being offered but, for example, some studies found that it was necessary to carefully select the activities available for older people and very young children (0–5years) to engage with together so as to ensure the generations were able and willing to mix, others found they needed to make sure there was a choice of activities available, whilst others working with older young people (19–30) found that sometimes extra preparation was needed for those groups to feel confident or ready to engage with their older adult counterparts. Approximately 55 studies explored mechanisms underlying the intervention being studied. Elements such as valuing interactions that incorporate learning and insights in both generations (Lane, 2016); how promoting positive experiences was key to developing meaningful and satisfying relationships (Kamei et al., 2021); how characteristics of either generation can impact on success/engagement, and how success/engagement in these interventions can impact on the characteristics of both generations. Sustainability factors were explored by 46 studies, these factors overlap with factors affecting implementation but also look forward towards resolving challenges for future interventions. Very few studies explored managing risk within the intervention (*n* = 7), and of those that did, the concerns were related to the circumstances where young people shared accommodation with older people or where young children entered an older person's setting like a day care centre or care home.

### 5.3 Risk of bias in included reviews

Risk of bias was not assessed as part of this EGM as per the protocol.

### 5.4 Additional dimensions (if applicable)

#### 5.4.1 Participant characteristics

We were able to identify studies that targeted specific participant characteristics, and these are described below.

##### Progress plus characteristics

Fifty‐one studies targeted children and young people with vulnerable characteristics. Of these, 6 involved minority groups (institutionalised children, those affected by race or cultural differences), 13 involved children and young people from low socioeconomic backgrounds, 2 involved those experiencing social isolation, 11 involved children with educational needs and 5 involved young people who were unemployed. Eighty‐eight studies targeted older people with vulnerable characteristics. Of these two involved minority groups (those affected by race or cultural differences), ten involved those from low socioeconomic backgrounds, five involved those experiencing social isolation and no interventions specifically involved older people who were unemployed. We did not identify any research that looked at other Progress Plus characteristics such as gender, LGBTQ, religion or place of residence.

##### Other important characteristics (discussed with the stakeholder advisory group)

Of the 51 studies that targeted children and young people with vulnerable characteristics, 6 involved those with mental health difficulties, 6 involved children with physical difficulties and 22 involved children and young people experiencing childhood adversity. Of the 88 studies that targeted older people with vulnerable characteristics, 14 involved those with mental health difficulties, 25 involved older people with physical difficulties and 49 involved older people with cognitive impairment.

Only 12 interventions involved participants with multiple vulnerability characteristics across the generations. For example, one intervention involved older people from a low income background (and some with additional physical health conditions) and young people with mental health problems (E. D. Jones et al., 2004); or young unemployed people and older people with a physical health condition (Schindler, 1992); or children with educational needs and older people with mental or physical health difficulties (Kamei et al., 2020) or where both generations shared the same vulnerability such as a physical health condition (Macmillan‐Smith 1999; Sherman, 1997); low SES (Alcock et al., 2011; Carney, 1985; Kerrigan & Stevenson, 1997; La Porte, 1999; Rogers, 1994); social isolation (Jackson et al., 2019); or multiple vulnerabilities (Barbosa et al., 2020).

#### 5.4.2 Setting

The intergenerational interventions identified in this EGM took place across at least 10 different settings described below (Table 3). The descriptions in 25 studies were unclear where (which setting) the intervention was conducted in and where ‘other’ is reported in the setting (*n* = 28) 10 are systematic reviews covering more than one setting, eight are interventions that used digital interventions such that the true ‘setting’ may be mixed or unclear, seven are interventions that took place in mixed settings, and three are interventions that were conducted in a holiday/retreat type setting. None of the studies were conducted in secure institutions.

**Table 3:** List of settings for interventions in included studies.

Setting	Number of studies
School	162
Community	135
Care Homes	110
Higher education	70
Day care centres for older people	31
Retirement community	31
Shared site facilities	31
Assisted living facilities	25
Hospital	7
Other	28

#### 5.4.3 Intervention focus

Approximately 25 different intervention activities (or focuses) were recorded in this EGM (Table 4). Some interventions involved multiple activities to engage the generations but others have specifically concentrated on one main approach. The most commonly reported activities were those that included sharing perspectives of being older (*n* = 200), in part reflecting the fact that many of these interventions have been designed to address negative stereotypes and perceptions of older or younger age groups. The limited number of evaluations of older and younger people sharing living accommodation (*n* = 9) possibly reflects the few examples of these types of innovations. One‐hundred and sixty‐four interventions also included other forms of interaction such as spending time together, helping with chores, helping more generally within a school environment and mentoring.

**Table 4:** Reported activities in intergenerational interventions.

Activity	Number of studies
Involved sharing perspectives of being an older or younger person/child	200
Interventions also included other forms of interaction such as spending time together, helping with chores, helping more generally within a school environment, and mentoring	164
Interventions used art and crafts to engage the generations together	154
Learning or sharing music	127
Involved play games together	109
Involved supporting children to learn to read	82
Involved students interacting with older people to improve their professional education and skills	68
Storytelling	66
Exercise	65
Learning or sharing history	59
Sharing meals together	58
Learning or sharing IT skills	51
Used drama	41
Dance	36
Cooking activities	36
Gardening activities	30
Joint trips, events and excursions	27
Sharing literature or learning literacy	21
Writing letters	14
Reminiscence	13
Learning or practicing a new language	12
Sharing science activities	12
Used environmental activities such as developing sustainable communities or forest school activities	11
Learning or helping with maths	10
Students or young people sharing accommodation with older people	9

#### 5.4.4 Bibliometric analysis

**Figure 6:**
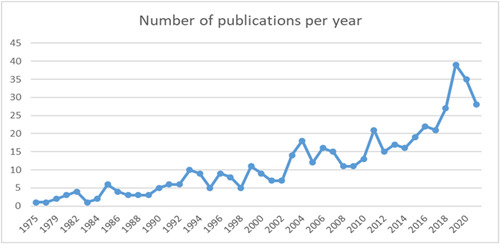
Number of publication per year.

In Figure 6 we can see there has been a steady increase in the number of studies evaluating intergenerational interventions published, with the first and single study published in 1975, to 35 in 2020. This may reflect a growing trend in evaluating these types of interventions and publishing the results, or an increase in the number of intergenerational interventions (Figure 6).

## 6 DISCUSSION

### 6.1 Summary of main results

This EGM presents the available evidence on non‐familial intergenerational interventions that involve direct contact or interaction between younger and older people two generations apart (at least one skipped generation in between). Below we address the literature in accordance with our three research questions:

*RQ1—What is the volume, nature and diversity of research on, and evaluation of, intergenerational practice and learning?*

We found a substantial amount of research literature (*n* = 500 studies) in this area of varying design, setting, focus, content and outcome. There are 26 systematic reviews, 38 RCTs, 198 non‐RCTs, 227 qualitative studies, 125 observational studies and 82 mixed methods studies. Most interventions include in this map are at Level 5 (*n* = 284), Level 6 (*n* = 155) or Level 7 (*n* = 35) of the Intergenerational engagement scale (Kaplan, 2004)—these are interventions with the most/deepest intergenerational engagement structures in place, and that may offer more lasting impacts on participant outcomes and be more sustainable and integrated in the future. These interventions take place in a range of settings assisted living facilities (*n* = 25), care homes (*n* = 110), community setting (*n* = 135), day care centres for older people (*n* = 31), hospital (*n* = 7), retirement community (*n* = 31), school (*n* = 162), university or higher education institution (*n* = 70) and shared site facilities (*n* = 31). The most commonly reported outcomes amongst the studies in this EGM are attainment and knowledge (*n* = 165), agency (*n* = 174), mental wellbeing (*n* = 224), attitudes towards the other generation (*n* = 283) and intergenerational interactions (*n* = 196), although mental health, physical health, loneliness and social isolation are also commonly reported. Interventions that involve people with vulnerability characteristics are also identified within this EGM.

*RQ2—What approaches have been used to deliver intergenerational activities and programmes that may be relevant to providing such services during and in the subsequent recovery from the COVID‐19 pandemic?*

The interventions themselves report using at least one of 25 different activities as the focus for an intervention but in many occasions multiple activities are used Table 4. Some of these activities (*n* = 8) were conducted online which would enable these activities in particular to carry on amid a pandemic. Such activities included but were not limited to sharing learning or perspectives and gaming online or mentoring through videoconferencing or email, or letter writing. Some activities that can be conducted either online or in outside spaces may work for pandemic recovery periods such as gardening activities, physical exercise or leisure activities conducted outside, excursions or trips or environmental activities. Other activities that need direct in person contact through music, drama, arts and crafts might be more suited to non‐pandemic times.

*RQ3—What promising intergenerational activities and programmes have been developed and are being used but have not yet been subject to formal evaluation?*

We were unable to answer this research question first due to the amount of research literature identified and so we were unable search for news items that would have identified interventions that exist but do not yet have research evidence available for them. Secondly, the complexity of the interventions are that ‘named’ interventions are not common and so what is identified in the literature are combinations of activities rather than interventions with specific models and structures.

### 6.2 Areas of major gaps in the evidence

This EGM has highlighted approximately ten areas in which research evidence is lacking (evidence gaps):

1. Many of the included studies evaluated the impact of intergenerational interventions on only one of the generations, often measuring and reporting outcomes for older people only. This finding was a surprise to our stakeholders, particularly those involved in the delivery of intergenerational activities, since in their experience benefits are often observed not only in terms of personal outcomes but also mutual or societal outcomes. Future research should consider how best to measure the broader impact of intergenerational activities.
2. Research evidence for interventions categorised as Levels 1–4 and 7 in Kaplans Intergenerational Engagement Scale (Kaplan, 2004). This could be due to interventions in Levels 1–2 being excluded from this EGM as they do not involve direct/personal contact or that research on interventions at these Levels (1–4) is less frequently conducted. Level 7 interventions are larger scale and more complex to study and therefore may not have been tested or implemented so frequently.
3. Mental health outcomes in children and young people—whilst there are some studies looking at this outcome (*n* = 14) the general lack of studies measuring this outcome seems to be at odds with the amount of intergenerational research available more generally
4. Loneliness and social isolation in children and young people, both as an outcome (*n* = 14) but also as a targeted characteristic (*n* = 2)
5. Peer interactions (*n* = 11), physical health outcomes (*n* = 10) and health promotion (*n* = 9) in children and young people
6. Health promotion in older people (*n* = 19)
7. Outcomes centred on others, for example, carers, care givers… mental health (*n* = 0), mental wellbeing (*n* = 12) and attitudes (*n* = 21)
8. Economic outcomes (*n* = 3)
9. Process outcomes—such as those related to managing risk (*n* = 7)
10. Adverse/unexpected outcomes whilst often reported (*n* = 47) are not consistently measured or reliably reported.

### 6.3 Potential biases in the mapping process

#### 6.3.1 Limitations of the EGM

Due to the amount of research literature available we did not include news items describing intergenerational activities and programmes even if they reported innovative interventions not otherwise represented within the evidence base. Whilst we recognise that this might mean the EGM is not comprehensive in terms of capturing all the existing intergenerational interventions, we are confident the EGM captures all the robust research in this area.

By nature of our inclusion criteria that specifies that ‘Any intervention that seeks to bring older and younger people together to intentionally with the purpose of interacting’, the EGM does not include interventions at level 1‐2 where there is no direct contact between the generations. This does not mean that these types of interventions are unlikely to have an impact but they are not the focus of our research interest.

We did not conduct quality appraisal of the research studies identified. We deemed this an appropriate approach as we wanted the EGM to be as comprehensive as possible in capturing the research picture without being confusing for the viewer (quality appraisal of different study designs would have been difficult to present in the EGM without oversimplifying the appraisal, which would undermine the usefulness of the information). The subsequent reviews that involve the use of this research map and that focus on intervention effectiveness, should ensure that quality appraisal is undertaken before making recommendations with regard to policy and practice.

Whilst the design of our framework may have limitations (other approaches may have been possible)—the design of our framework was led by the stakeholders. We used a framework that they were familiar with and is used by major intergenerational organisations—we were keen to use a framework that made sense to the people who we hoped would use the map. The level of engagement in an intervention was also seen to be a key driver for successful interventions and is also an indicator of the potential resource level required for implementation which may be helpful for some users. We felt that using the aims of an intervention would have been difficult to capture in the space of a map and would have been complex as interventions may have more than one aim. This might have made the map more difficult for users to access.

#### 6.3.2 Stakeholder engagement throughout the EGM process

We liaised with our stakeholders to confirm the details of the protocol before submitting this to Campbell. We were unable to meet with our stakeholders in person and conducted our first meeting online in one large group. At this meeting it was decided that subsequent meetings would be better conducted over two events within the same week to enable some flexibility in attendance and to ensure the meeting could be better facilitated for all attendees. At any point if any stakeholders could not attend the planned meetings they were given the opportunities to have one‐to‐one meetings with one of the project team or to share their thoughts and feedback over email. Stakeholders were also consulted about the structure of the EGM and how best to capture the outcomes they thought were important as well as the outcomes actively reported in the research. Two stakeholders have not engaged with the project so far but we hope to reconnect with them in the next stages. Details of the two meetings are in Table 5.

**Table 5:** Stakeholder engagement.

Meeting	No. of attendees	Content	Impact on the EGM/research
Stakeholder meeting 1	26 July 2021 (20 researchers, providers, commissioners, third sector and public perspectives represented)	– Introduction to the project (Jo Thompson Coon) – What are intergenerational activities? (Ellie Robinson‐Carter) – What is an evidence gap map? (Fiona Campbell) – Small group discussion in break out rooms to answer (using Jamboard):	Discussion and stakeholder contributions to the jamboard:
			– Enabled the research team to understand what type of intergenerational interventions there are and are likely to be identified in research. – Helped to inform the EGM about the outcomes that were important to capture and incorporate in the framework.
	Individual meetings arranged where possible/necessary		
		Q1: What are intergenerational activities? Do you know of any? What has been your experience of them?	
		Q2: What are the potential positive and negative outcomes that can come from intergenerational activities and what do you feel should be measured?	
Stakeholder meeting 2	Held over two meetings:	– Welcome and Introductions (Jo Thompson Coon) – Project update (Rebecca Whear and Morwenna Rogers) – numbers of screening and coding, initial map – Purpose of meeting (Rebecca Whear) – share what we have done so far, share map, explore it and think about the kinds of questions it raises but particularly thinking about research questions for the two reviews that we will be conducting as a result of this mapping exercise. – Present the map (Fiona Campbell) – – Any questions about the map? – Discuss potential questions for next reviews	Discussion and stakeholder contributions helped to:
	27 Sept (17 researchers, providers, commissioners and third sector perspectives represented)		
			– Understand how the EGM was interpreted and how its presentation could be improved – Helped to understand what the most useful next steps would be – Helped to determine the most relevant research questions for the second stage of the project
	28 Sept (16 researchers, commissioners, third sector and public perspectives represented)		

## 7 AUTHORS’ CONCLUSIONS

### 7.1 Implications for research, practice and/or policy

Based on the research identified in this EGM the implications for research are:

- A need to explore gaps in terms of promising interventions not yet formally evaluated.
- Further primary research needs to build on the evidence for existing interventions exploring a more consistent set of outcomes relevant to both generations engaged in the intervention. This should include the wider impact of the intervention on their families and/or carers and the wider community.
- More primary research is needed on mental health and the mental wellbeing of children and young people, and also loneliness and social isolation in both generations.
- Further primary research should also focus on issues with regard to intervention implementation and sustainability including economic outcomes so that policy makers and commissioners as well as service providers can make better informed decisions as to what intervention might work well and be sustainable for the community with which they are working.
- Further research needs to be conducted on Level 7 type interventions in which the interactions between the generations are built into the community and part of every‐day communication, interaction and general living, with the potential thereby for demonstrating lasting positive impacts for everyone involved. These interventions could potentially be more costly and therefore decision‐makers need to be confident about the individual, social, economic and community benefits (as well as costs).

Implications for policy are:

- Uncertain in many circumstances because much of the available research does not currently tell us what the impact of the interventions are on both generations (i.e., where there may be positive outcomes for one generation there is a need to be mindful of the outcomes experienced by the other generation). Research that explores the outcomes of an intervention for only one generation need to be further explored before being implemented.
- The research on this topic is gradually increasing, and systematic reviews will be important to determine how and why interventions are or are not beneficial. However, the primary research area needs to build more cohesively so that the findings can be comparable and avoid research waste.
- The EGM presented here will nevertheless be a useful resource for decision‐makers allowing them to explore the evidence with regard to the different interventions that may be relevant to their population needs and the settings or resources available to them.

## CONTRIBUTIONS OF AUTHORS

Content: ERC is a socially engaged creative practitioner and consultant based in Plymouth, founder of The Photobook Project and Project Manager at The Sensory Trust where she works on the dementia and intergenerational project Creative Spaces. This project uses nature and outdoor spaces to encourage older people with dementia to become more active, build social networks and foster independence. Previously she founded the multi‐award winning Penryn Memory Café and led a memory café in York for 2 years whilst at University. She has recently completed the International Certificate in Intergenerational Practice provided by Generations Working Together and the University of Granada. SC is Commissioning Manager at NHS Kernow Clinical Commissioning Group and has an interest in the role of intergenerational programmes and activities in health and social care. RS is an advanced public health specialist at Cornwall Council with an interest in the role of intergenerational programmes and activities in health and social care specifically in relation to the mental health of older adults. JB is an expert in the mental and social wellbeing of children and young people and also has expertise in evidence synthesis methodology.

EGM methods: JTC is an expert in evidence synthesis and health policy research. She is co‐chair and editor of the Ageing Group of the Campbell Library and co‐director of the Cochrane Campbell Global Ageing Partnership. RW is an expert in evidence synthesis methods. FC is editor of the Children and Adolescent Group of the Campbell Collaboration. She has over 20 years of experience in evidence synthesis and leads a short course in scoping, mapping and EGM reviews.

Information retrieval: MR is an information specialist with experience in health services research, methods editor for the Ageing Group of the Campbell Library and a member of the Campbell Information Retrieval Methods Group. AS is a Senior Information Specialist, with extensive experience of literature searching and information management for systematic reviews and other types of evidence syntheses on a wide range of topics, including integrated care, art therapy and quality of life. AS is the joint lead of a module on systematically reviewing the research literature for postgraduate students, and the joint author of the textbook *Systematic Approaches to a Successful Literature Review*, 2nd Edition published by Sage in 2016. Anthea is also the Reviews Editor for *Health Information and Libraries Journal*.

## DECLARATIONS OF INTEREST

ERC, members of our advisory group and members of the Only Connect steering group are involved in the delivery of intergenerational activities and programmes.

## PLANS FOR UPDATING THE EGM

Once completed the evidence gap map will be updated as resources permit.

## DIFFERENCES BETWEEN PROTOCOL AND REVIEW

Due to the substantial amount of research literature found we did not include news items describing intergenerational activities and programmes if they reported innovative interventions not otherwise represented within the evidence base (as in the protocol). However, this will be conducted in the subsequent reviews directly related to this EGM.

Due to the amount of research literature found we limited our additional searches (forwards and backwards citation chasing) as follows: we carried out backward citation chasing on the included studies within identified systematic reviews specifically looking for RCTs and systematic reviews not already included in the EGM; we did not check the citations of older key papers (forward citation chasing); we identified one key journal the Journal of Intergenerational Relationships and hand‐search the contents; we did not conduct the horizon scanning process (we will search Nexus for relevant international news articles about intergenerational practices and Google for relevant reports, blogs, news articles and links to other relevant organisations) mentioned in the protocol but expect to conduct that in subsequent reviews.

In addition to the filters mentioned in the protocol additional amendments were made to include the following:

– Characteristics of the participants: Childhood Adversity, Age category, Disability (physical heath difficulties), Mental health difficulties, Low socioeconomic status, Minority groups, Social isolation, Unemployed, Educational needs, Cognitive impairment.
– Contextual factors: Setting and Country.
– Study design factors.
– Focus of the interventions: Education, Art and craft, Music, Interaction, Cooking, Dance, Drama, Environmental activities, Exercise, Gardening, History, IT, Language, Letter writing, Literature, Living together, Maths, Playing games, Professional education, Reading, Reminiscence, Science activities, Sharing meals, Sharing perspectives (of being and older person/a child/young person), Story telling, Trips and excursions and Other.

## SOURCES OF SUPPORT

**Internal sources**

• No sources of support provided

**External sources**

- National Institute for Health Research (NIHR) Evidence Synthesis Programme NIHR 133097 and NIHR 133172, UK

The evidence gap map is funded by the National Institute for Health Research (NIHR) Evidence Synthesis Programme NIHR 133097 and NIHR 133172 and supported by the National Institute for Health Research (NIHR) Applied Research Collaboration South West Peninsula. The views expressed are those of the author(s) and not necessarily those of the NIHR or the Department of Health and Social Care.

## Supporting information

Supplementary Information

Supplementary Information

## References

[cl21306-bib-0002] OTHER REFERENCES

[cl21306-bib-0003] ADDITIONAL REFERENCES

[cl21306-bib-0004] Aday, R. H. , Aday, K. L. , Arnold, J. L. , & Bendix, S. L. (1996). Changing children's perceptions of the elderly. Gerontology & Geriatrics Education, 16(3), 37–51. 10.1300/J021v16n03_04 23621412

[cl21306-bib-0005] Alcock, C. L. , Camic, P. M. , Barker, C. , Haridi, C. , & Raven, R. (2011). Intergenerational practice in the community: A focused ethnographic evaluation. Journal of Community & Applied Social Psychology, 21(5), 419–432. 10.1002/casp.1084

[cl21306-bib-0006] Barbosa, M. R. , Campinho, A. , & Silva, G. (2020). “give and receive”: The impact of an intergenerational program on institutionalized children and older adults. Journal of Intergenerational Relationships, 19(3), 283–304. 10.1080/15350770.2020.1742844

[cl21306-bib-0007] Brown, C. , & Henkin, N. (2014). Building communities for all ages: Lessons learned from an intergenerational community‐building initiative. Journal of Community & Applied Social Psychology, 24(1), 63–68. 10.1002/casp.2172

[cl21306-bib-0008] Carney, J. M. (1985). Senior citizens as paraprofessionals in a public elementary school (intergenerational, self‐concept). Oklahoma State University.

[cl21306-bib-0009] Diachun, L. L. , Ivers, N. , Johnstone, J. , Nayot, D. , & Dumbrell, A. C. (2007). More to life than MacGyver and crocheting”: Medical students respond to a recreational, intergenerational event. Canadian Journal of Geriatrics, 10(2), 53–59.

[cl21306-bib-0010] Department of Health . (2005). Independence, well‐being and choice:Our vision for the future of social care for adults in England.

[cl21306-bib-0011] Doll, G. , & Bolender, B. (2010). Age to age: Resident outcomes from a kindergarten classroom in the nursing home. Journal of Intergenerational Relationships, 8(4), 327–337.

[cl21306-bib-0012] Dumbrell, A. C. , Durst, M. A. , & Diachun, L. L. (2007). White coats meet grey power: students and seniors respond to an “intergenerational gala”. Journal of the American Geriatrics Society, 55(6), 948–954. 17537099 10.1111/j.1532-5415.2007.01189.x

[cl21306-bib-0013] Edström, M. (2018). Visibility patterns of gendered ageism in the media buzz: a study of the representation of gender and age over three decades. Feminist media. Studies, 18(1), 77–93. 10.1080/14680777.2018.1409989

[cl21306-bib-0014] Endnote X8 . (2017). [Computer program]. Clarivate.

[cl21306-bib-0015] EPPI‐Mapper . (2022). Version 2.1.0 [Computer program]. Digital Solution Foundry and EPPI‐Centre. EPPI‐Centre, UCL Social Research Institute, University College London.

[cl21306-bib-0016] Gardner, P. , & Alegre, R. (2019). “Just like us”: Increasing awareness, prompting action and combating ageism through a critical intergenerational service learning project. Educational Gerontology, 45(2), 146–158. 10.1080/03601277.2019.1584976

[cl21306-bib-0017] Generations Working Together . (2019). Generations Working Together Corporate Plan 2020‐2025 Towards an intergenerationally connected Scotland. Generations Working Together.

[cl21306-bib-0018] Hock, N. , & Mickus, M. (2019). An intergenerational residential model for elders and students. Journal of Intergenerational Relationships, 17(3), 380–387. 10.1080/15350770.2019.1617605

[cl21306-bib-0019] Howell, B. M. , Redmond, L. C. , & Wanner, S. (2021). “I learned that I am loved”: Older adults and undergraduate students mutually benefit from an interprofessional service‐learning health promotion program. Gerontology & Geriatrics Education, 42(2), 252–267. 10.1080/02701960.2020.1791104 32654625

[cl21306-bib-0020] Jackson, E. , Allen, I. E. , Perissinotto, C. , McHugh Power, J. , Lawlor, B. , Miller, B. L. , & Bell, P. (2019). Impact on young adults from participation in the Multimodal Intergenerational Social Contact Intervention Pilot for Creative Engagement (MISCI‐PCE): AN intergenerational dementia risk reduction strategy. Alzheimer's and Dementia, 15(7), 1206. 10.1016/j.jalz.2019.06.3637

[cl21306-bib-0021] Jones, E. D. , Herrick, C. , & York, R. F. (2004). An intergenerational group benefits both emotionally disturbed youth and older adults. Issues in Mental Health Nursing, 25(8), 753–767. 15545241 10.1080/01612840490506329

[cl21306-bib-0022] Jones, R. L. (2011). Imagining old age. In J. Katz , S. Peace , & S. Spurr (Eds.), Adult lives: A life course perspective (pp. 18–26). Policy Press.

[cl21306-bib-0023] Kamei, T. , Meguro, S. , Yamamoto, Y. , & Kanamori, T. (2020). St. Luke's Intergenerational Day Program; Nagomi‐no‐kai (Harmonized Program): Program profile. Journal of Intergenerational Relationships, 18(1), 106–112. 10.1080/15350770.2020.1709952

[cl21306-bib-0024] Kamei, T. , Yamamoto, Y. , Kanamori, T. , & Tomioka, S. (2021). A prospective longitudinal mixed methods study of program evaluation in an intergenerational program: Intergenerational interactions and program satisfactions involving non‐frail, frail, cognitively impaired older adults, and school aged‐children. Journal of Intergenerational Relationships, 20(1), 60–80. 10.1080/15350770.2020.1853650

[cl21306-bib-0025] Kaplan, M. S. (2004). Toward an intergenerational way of life. Journal of Family and Consumer Sciences, 96(2), 5.

[cl21306-bib-0026] Kerrigan, J. , & Stevenson, N. C. (1997). Behavioral study of youth and elders in an intergenerational horticultural program. Activities. Adaptation & Aging, 22(3), 141–153. 10.1300/J016v22n03_02

[cl21306-bib-0027] Kilaberia, R. , & Ratner, E. (2018). Live‐in “strangers”: An experiential account of gerontology educational immersion in senior housing. Gerontology & Geriatrics Education, 39(1), 86–103. 10.1080/02701960.2016.1188809 27637000

[cl21306-bib-0028] Kingman, D. (2016). *Generations apart? The growth of age segregation in England and Wales*.

[cl21306-bib-0029] Krzeczkowska, A. , Spalding, D. M. , McGeown, W. J. , Gow, A. J. , Carlson, M. C. , & Nicholls, L. A. B. (2021). A systematic review of the impacts of intergenerational engagement on older adults’ cognitive, social, and health outcomes. Ageing Research Reviews, 71, 101400. 34237435 10.1016/j.arr.2021.101400

[cl21306-bib-0030] La Porte, A. M. (1999). Building community through intergenerational art education. Resources in Education, ERIC. Indiana University. Paper presented at the Annual Meeting of the National Art Education Association, Washinton D.C. March 24‐28, 1999.

[cl21306-bib-0031] Labit, A. , & Dubost, N. (2016). Housing and ageing in France and Germany: The intergenerational solution. Housing Care and Support, 19(2), 45–54.

[cl21306-bib-0032] Lane, K. (2016). “Are you going to come and see us again soon?” An intergenerational event between stroke survivors and school‐children. Quality in Ageing and Older Adults, 17(4), 246–252.

[cl21306-bib-0033] Laurence, J. (2016). Wider‐community segregation and the effect of neighbourhood ethnic diversity on social capital: An investigation into Intra‐Neighbourhood trust in Great Britain and London. Sociology, 51(5), 1011–1033. 10.1177/0038038516641867 28989199 PMC5603975

[cl21306-bib-0034] Lytle, A. , Nowacek, N. , & Levy, S. R. (2020). Instapals: Reducing ageism by facilitating intergenerational contact and providing aging education. Gerontology & Geriatrics Education, 41(3), 308–319. 10.1080/02701960.2020.1737047 32129732

[cl21306-bib-0035] MacMillan‐Smith, L. C. (1999). *Intergenerational echange: Effects on Attitude of Deaf Youth Participating in a Programwith Deaf Older Adults*. University of Michigan‐Flint.

[cl21306-bib-0036] Nazroo, J. , Greenwad, l. I. , Bajekal, M. , & Lewis, J. (2005). Ethnic inequalities in quality of life at older ages: Subjective and objective components. ESRC.

[cl21306-bib-0037] O'Neill, J. , Tabish, H. , Welch, V. , Petticrew, M. , Pottie, K. , Clarke, M. , Evans, T. , Pardo Pardo, J. , Waters, E. , White, H. , & Tugwell, P. (2014). Applying an equity lens to interventions: using PROGRESS ensures consideration of socially stratifying factors to illuminate inequities in health. Journal of Clinical Epidemiology, 67(1), 56–64. 10.1016/j.jclinepi.2013.08.005 24189091

[cl21306-bib-0038] Office for National Statistics . (2021). Loneliness ‐What characteristics and circumstances are associated with feeling lonely? Analysis of characteristics and circumstances associated with loneliness in England using the Community Life Survey, 2020‐2021.

[cl21306-bib-0039] Page, M. J. , McKenzie, J. E. , Bossuyt, P. M. , Boutron, I. , Hoffmann, T. C. , Mulrow, C. D. , Shamseer, L. , Tetzlaff, J. M. , Akl, E. A. , Brennan, S. E. , Chou, R. , Glanville, J. , Grimshaw, J. M. , Hróbjartsson, A. , Lalu, M. M. , Li, T. , Loder, E. W. , Mayo‐Wilson, E. , McDonald, S. , … Moher, D. (2021). The PRISMA 2020 statement: an updated guideline for reporting systematic reviews. BMJ, 372, n71. 10.1136/bmj.n71 33782057 PMC8005924

[cl21306-bib-0040] Rogers, A. M. (1994). Intergenerational mentoring: Benefits and barriers for elder mentors [PhD, Temple University].

[cl21306-bib-0041] Rosa‐Hernandez, G. B. , Murray, C. M. , & Stanley, M. (2020). An intergenerational playgroup in an Australian residential aged‐care setting: A qualitative case study. Health & Social Care in the Community, 1–10. 10.1111/hsc.13149 32852104

[cl21306-bib-0042] Schindler, D. L. (1992). Intergenerational programming: A confluence of interests between the frail elderly and urban youth [PhD, Portland State University].

[cl21306-bib-0043] Sherman, A. (1997). A case study of intergenerational relations through dance with profoundly deaf individuals. Journal of Gerontological Social Work, 28(1–2), 113–123. 10.1300/J083v28n01_14

[cl21306-bib-0044] Skropeta, C. M. , Colvin, A. , & Sladen, S. (2014). An evaluative study of the benefits of participating in intergenerational playgroups in aged care for older people. BMC Geriatrics, 14, 109. 10.1186/1471-2318-14-109 25292218 PMC4197292

[cl21306-bib-0045] Surkalim, D. L. , Luo, M. , Eres, R. , Gebel, K. , van Buskirk, J. , Bauman, A. , & Ding, D. (2022). The prevalence of loneliness across 113 countries: systematic review and meta‐analysis. BMJ, 376, e067068. 10.1136/bmj-2021-067068 35140066 PMC8826180

[cl21306-bib-0046] Thomas, J. , Graziosi, S. , Brunton, J. , Ghouze, Z. , O'Driscoll, P. , & Bond, M. (2022). EPPI‐Reviewer: Advanced software for systematic reviews, maps and evidence synthesis [Computer program]. EPPI‐Centre, UCL Social Research Institute, University College London.

[cl21306-bib-0047] Thompson‐Coon, J. , Campbell, F. , Sutton, A. , Whear, R. , Rogers, M. , Barlow, J. , Carter, E. R. , Sharpe, R. , Cohen, S. , & Wolstenholme, L. (2022). PROTOCOL: Intergenerational interventions and their effect on social and mental wellbeing of both children and older people—A mapping review and evidence and gap map. Campbell Systematic Reviews, 18(2), e1235. 10.1002/cl2.1235 PMC9107595 36911353

[cl21306-bib-0048] UNICEF & WHO . (2020). Investing in our future: A comprehensive agenda for the health and well‐being of children and adolescents. WHO.

[cl21306-bib-0049] United for all Ages . (2017). *A country for all ages: Ending age apartheid in Brexit Britain*.

[cl21306-bib-0050] Vasil, L. , & Wass, H. (1993). Portrayal of the elderly in the media: A literature review and implications for educational gerontologists. Educational Gerontology, 19(1), 71–85. 10.1080/0360127930190107

[cl21306-bib-0051] Vitman, A. , Iecovich, E. , & Alfasi, N. (2013). Ageism and social integration of older adults in their neighborhoods in Israel. The Gerontologist, 54(2), 177–189. 10.1093/geront/gnt008 23463803

[cl21306-bib-0052] Werner, D. , Teufel, J. , Holtgrave, P. L. , & Brown, S. L. (2012). Active generations: an intergenerational approach to preventing childhood obesity. Journal of School Health, 82(8), 380–386. 10.1111/j.1746-1561.2012.00713.x 22712675

[cl21306-bib-0053] White, H. , Welch, V. , Pigott, T. , Marshall, Z. , Snilstveit, B. , Mathew, C. , & Littell, J. (2018). Campbell Collaboration checklist for evidence and gap maps: Conduct standards. Campbell Collaboration.

