# Supplementary material for: Non‐familial intergenerational interventions and their impact on social and mental wellbeing of both younger and older people—A mapping review and evidence and gap map
Source: Campbell Syst Rev. 2023 Feb 16;19(1):e1306. doi: 10.1002/cl2.1306 (PMC9934919; doi:10.1002/cl2.1306)
Supplement: Supplementary file 1 — Supplementary Information [file CL2-19-e1306-s002.docx]

Appendices

1 Search strategies

[Enter text here]Database Search Strategies

MEDLINE via OvidSp

Database: Ovid MEDLINE(R) ALL <1946 to July 21, 2021>

Search Strategy:

--------------------------------------------------------------------------------

1 Intergenerational Relations/ (4146)

2 (intergenerational or inter generational).ti,ab. (6528)

3 cross age.ti,ab. (109)

4 across generation*.ti,ab. (2061)

5 cross generation*.ti,ab. (438)

6 ((generations or different age groups or all ages or all age groups or mixed ages or mixed age groups or (old* adj2 young*)) adj5 (together or social engagement or connecting)).ti,ab. (257)

7 (intergenerational adj2 (program* or intervention*)).ti. (71)

8 or/1-6 (11703)

9 adolescent/ or child/ or child, preschool/ (3143466)

10 child*.ti,ab. (1463735)

11 (young adj (person or people or male* or female*)).ti,ab. (54389)

12 (youth* or teen*).ti,ab. (110196)

13 young offender*.ti,ab. (499)

14 (school and pupil*).ti,ab. (4871)

15 preschooler*.ti,ab. (7441)

16 student*.ti,ab. (304578)

17 (girl or girls or boy or boys).ti,ab. (240923)

18 or/9-17 (3888702)

19 exp Aged/ (3276163)

20 dementia.ti,ab. (115820)

21 alzheimer*.ti,ab. (156770)

22 old*.ti,ab. (1566913)

23 elderly.ti,ab. (258523)

24 geriatric.ti,ab. (44411)

25 (residents or resident).ti,ab. (170641)

26 (elder or elders).ti,ab. (16913)

27 (retired or retiree*).ti,ab. (7895)

28 veteran*.ti,ab. (38644)

29 grandfriend*.ti,ab. (0)

30 seniors.ti,ab. (7890)

31 (senior adj citizen*).ti,ab. (1548)

32 (centarian* or centenarian* or nonagenarian* or octagenarian* or octogenarian* or sexagenarian* or septuagenarian*).ti,ab. (6890)

33 or/19-32 (4773325)

34 program*.ti,ab. (937612)

35 activit*.ti,ab. (3230552)

36 interaction*.ti,ab. (1398324)

37 (project or projects).ti,ab. (220965)

38 intervention*.ti,ab. (1099124)

39 initiative*.ti,ab. (95427)

40 scheme.ti,ab. (104838)

41 visit*.ti,ab. (255175)

42 reading.ti,ab. (118535)

43 (play or playing or playtime).ti,ab. (759787)

44 music.ti,ab. (17586)

45 boardgame*.ti,ab. (3)

46 games.ti,ab. (16559)

47 voluntary.ti,ab. (64481)

48 volunteering.ti,ab. (2125)

49 mentor*.ti,ab. (17791)

50 or/34-49 (7060335)

51 Homes for the Aged/ (14400)

52 Nursing Homes/ (36493)

53 care home*.ti,ab. (4517)

54 nursing home*.ti,ab. (31757)

55 residential care.ti,ab. (3647)

56 ((senior or elderly or old) adj day care).ti,ab. (46)

57 ((hospital* or ward) adj3 geriatric*).ti,ab. (3384)

58 community.ti,ab. (529188)

59 (sheltered adj (housing or accommodation)).ti,ab. (257)

60 (retirement adj (home* or village* or complex*)).ti,ab. (476)

61 (abbeyfield or almshouse*).ti,ab. (65)

62 (geriatric adj (institution* or care)).ti,ab. (2026)

63 assisted living.ti,ab. (2324)

64 own home.ti,ab. (1205)

65 (preschool or preschools).ti,ab. (26214)

66 playgroup*.ti,ab. (142)

67 (school or schools or college*).ti,ab. (398572)

68 (nursery or nurseries).ti,ab. (11824)

69 kindergarten*.ti,ab. (7051)

70 play setting*.ti,ab. (90)

71 (child care setting* or childcare setting*).ti,ab. (423)

72 (child* adj2 day care).ti,ab. (1110)

73 or/51-72 (987221)

74 8 and 18 and 33 and 50 (955)

75 8 and 18 and 50 and 73 (809)

76 8 and 33 and 50 and 73 (516)

77 7 or 74 or 75 or 76 (1567)

EMBASE via OvidSp

Database: Embase <1974 to 2021 July 26>

Search Strategy:

--------------------------------------------------------------------------------

1 (intergenerational or inter generational).ti,ab. (7195)

2 cross age.ti,ab. (116)

3 across generation*.ti,ab. (2274)

4 cross generation*.ti,ab. (503)

5 ((generations or different age groups or all ages or all age groups or mixed ages or mixed age groups or (old* adj2 young*)) adj5 (together or social engagement or connecting)).ti,ab. (315)

6 (intergenerational adj (program* or intervention*)).ti. (63)

7 or/1-5 (9973)

8 child*.ti,ab. (1839835)

9 (young adj (person or people or male* or female*)).ti,ab. (73378)

10 (youth* or teen*).ti,ab. (139345)

11 young offender*.ti,ab. (657)

12 (school and pupil*).ti,ab. (6479)

13 preschooler*.ti,ab. (8618)

14 student*.ti,ab. (398095)

15 (girl or girls or boy or boys).ti,ab. (319264)

16 or/8-15 (2474880)

17 dementia.ti,ab. (168681)

18 alzheimer*.ti,ab. (214580)

19 old*.ti,ab. (2218941)

20 elderly.ti,ab. (365236)

21 geriatric.ti,ab. (67443)

22 (residents or resident).ti,ab. (224475)

23 (elder or elders).ti,ab. (24475)

24 (retired or retiree*).ti,ab. (11390)

25 veteran*.ti,ab. (51001)

26 grandfriend*.ti,ab. (0)

27 seniors.ti,ab. (10783)

28 (senior adj citizen*).ti,ab. (1955)

29 (centarian* or centenarian* or nonagenarian* or octagenarian* or octogenarian* or sexagenarian* or septuagenarian*).ti,ab. (9976)

30 or/17-29 (2985765)

31 (Intergenerational adj5 program*).ti,ab. (252)

32 (Intergenerational adj5 activit*).ti,ab. (73)

33 interaction*.ti,ab. (1586512)

34 (Intergenerational adj5 (project or projects)).ti,ab. (51)

35 (intergenerational adj5 intervention*).ti,ab. (133)

36 initiative*.ti,ab. (135248)

37 scheme.ti,ab. (118860)

38 visit*.ti,ab. (411575)

39 reading.ti,ab. (137382)

40 (play or playing or playtime).ti,ab. (958236)

41 music.ti,ab. (21767)

42 boardgame*.ti,ab. (6)

43 games.ti,ab. (19999)

44 voluntary.ti,ab. (81898)

45 volunteering.ti,ab. (2684)

46 mentor*.ti,ab. (23424)

47 or/31-46 (3299598)

48 exp home for the aged/ (11191)

49 nursing home/ (55019)

50 care home*.ti,ab. (5719)

51 nursing home*.ti,ab. (40613)

52 residential care.ti,ab. (4537)

53 ((senior or elderly or old) adj day care).ti,ab. (51)

54 ((hospital* or ward) adj3 geriatric*).ti,ab. (5394)

55 community.ti,ab. (658292)

56 (sheltered adj (housing or accommodation)).ti,ab. (348)

57 (retirement adj (home* or village* or complex*)).ti,ab. (633)

58 (abbeyfield or almshouse*).ti,ab. (50)

59 (geriatric adj (institution* or care)).ti,ab. (2776)

60 assisted living.ti,ab. (3069)

61 own home.ti,ab. (1906)

62 (preschool or preschools).ti,ab. (30630)

63 playgroup*.ti,ab. (184)

64 (school or schools or college*).ti,ab. (574672)

65 (nursery or nurseries).ti,ab. (12133)

66 kindergarten*.ti,ab. (7954)

67 play setting*.ti,ab. (100)

68 (child care setting* or childcare setting*).ti,ab. (500)

69 (child* adj2 day care).ti,ab. (1243)

70 or/48-69 (1301852)

71 7 and 16 and 30 and 47 (382)

72 7 and 16 and 47 and 70 (411)

73 7 and 30 and 47 and 70 (270)

74 6 or 71 or 72 or 73 (707)

Psyc||NFO via OvidSp

Database: APA PsycInfo <1806 to July Week 2 2021>

Search Strategy:

--------------------------------------------------------------------------------

1 (intergenerational or inter generational).ti,ab. (10856)

2 cross age.ti,ab. (425)

3 across generation*.ti,ab. (1804)

4 cross generation*.ti,ab. (579)

5 ((generations or different age groups or all ages or all age groups or mixed ages or mixed age groups or (old* adj2 young*)) adj5 (together or social engagement or connecting)).ti,ab. (216)

6 (intergenerational adj2 (program* or intervention*)).ti. (183)

7 or/1-5 (13230)

8 child*.ti,ab. (718266)

9 (young adj (person or people or male* or female*)).ti,ab. (36091)

10 (youth* or teen*).ti,ab. (124134)

11 young offender*.ti,ab. (1382)

12 (school and pupil*).ti,ab. (12463)

13 preschooler*.ti,ab. (13210)

14 student*.ti,ab. (530181)

15 (girl or girls or boy or boys).ti,ab. (105641)

16 or/8-15 (1304003)

17 *retirement/ (3966)

18 *nursing home residents/ (1372)

19 dementia.ti,ab. (65851)

20 alzheimer*.ti,ab. (62559)

21 (old adj (adult* or people or aged)).ti,ab. (4118)

22 elderly.ti,ab. (58939)

23 geriatric.ti,ab. (14583)

24 (residents or resident).ti,ab. (47815)

25 (elder or elders).ti,ab. (10953)

26 (retired or retiree*).ti,ab. (4562)

27 veteran*.ti,ab. (22838)

28 pensioner*.ti,ab. (246)

29 grandfriend*.ti,ab. (2)

30 seniors.ti,ab. (7210)

31 (senior adj citizen*).ti,ab. (1063)

32 (centarian* or centenarian* or nonagenarian* or octagenarian* or octogenarian* or sexagenarian* or septuagenarian*).ti,ab. (740)

33 or/17-32 (242158)

34 (intergenerational adj10 program*).ti,ab. (610)

35 activit*.ti,ab. (447902)

36 interaction*.ti,ab. (326924)

37 (intergeneration adj10 (project or projects)).ti,ab. (0)

38 (intergenerational adj10 intervention*).ti,ab. (277)

39 initiative*.ti,ab. (44284)

40 scheme.ti,ab. (14753)

41 visit*.ti,ab. (55959)

42 reading.ti,ab. (115232)

43 (play or playing or playtime).ti,ab. (169593)

44 music.ti,ab. (31587)

45 boardgame*.ti,ab. (5)

46 games.ti,ab. (23001)

47 voluntary.ti,ab. (27777)

48 volunteering.ti,ab. (2920)

49 mentor*.ti,ab. (18031)

50 or/34-49 (1114650)

51 Nursing Homes/ (9029)

52 *retirement communities/ (282)

53 care home*.ti,ab. (1933)

54 nursing home*.ti,ab. (11930)

55 residential care.ti,ab. (3653)

56 ((senior or elderly or old) adj day care).ti,ab. (31)

57 ((hospital* or ward) adj3 geriatric*).ti,ab. (859)

58 (community and intergenerational).ti,ab. (1265)

59 (sheltered adj (housing or accommodation)).ti,ab. (134)

60 (retirement adj (home* or village* or complex*)).ti,ab. (270)

61 (abbeyfield or almshouse*).ti,ab. (43)

62 (geriatric adj (institution* or care)).ti,ab. (611)

63 assisted living.ti,ab. (1267)

64 own home.ti,ab. (633)

65 (preschool or preschools).ti,ab. (35382)

66 playgroup*.ti,ab. (241)

67 (school or schools or college*).ti,ab. (514342)

68 (nursery or nurseries).ti,ab. (4671)

69 kindergarten*.ti,ab. (18397)

70 play setting*.ti,ab. (294)

71 (child care setting* or childcare setting*).ti,ab. (538)

72 (child* adj2 day care).ti,ab. (437)

73 or/51-72 (571270)

74 7 and 16 and 33 and 50 (430)

75 7 and 16 and 50 and 73 (977)

76 7 and 33 and 50 and 73 (297)

77 6 or 74 or 75 or 76 (1307)

***************************

Database: Social Policy and Practice <202104>

Search Strategy:

--------------------------------------------------------------------------------

1 (intergenerational or inter generational).ti,ab. (1952)

2 cross age.ti,ab. (13)

3 across generation*.ti,ab. (105)

4 cross generation*.ti,ab. (33)

5 ((generations or different age groups or all ages or all age groups or mixed ages or mixed age groups or (old* adj2 young*)) adj5 (together or social engagement or connecting)).ti,ab. (96)

6 (intergenerational adj2 (program* or intervention*)).ti. (75)

7 or/1-5 (2077)

8 child*.ti,ab. (82146)

9 (young adj (person or people or male* or female*)).ti,ab. (25148)

10 (youth* or teen*).ti,ab. (13750)

11 young offender*.ti,ab. (1234)

12 (school and pupil*).ti,ab. (2759)

13 preschooler*.ti,ab. (99)

14 student*.ti,ab. (10175)

15 (girl or girls or boy or boys).ti,ab. (3953)

16 or/8-15 (109065)

17 dementia.ti,ab. (13307)

18 alzheimer*.ti,ab. (3134)

19 old*.ti,ab. (51433)

20 elderly.ti,ab. (14212)

21 geriatric.ti,ab. (2515)

22 (residents or resident).ti,ab. (11232)

23 (elder or elders).ti,ab. (3684)

24 (retired or retiree*).ti,ab. (819)

25 veteran*.ti,ab. (473)

26 grandfriend*.ti,ab. (1)

27 seniors.ti,ab. (461)

28 (senior adj citizen*).ti,ab. (196)

29 (centarian* or centenarian* or nonagenarian* or octagenarian* or octogenarian* or sexagenarian* or septuagenarian*).ti,ab. (180)

30 or/17-29 (80130)

31 program*.ti,ab. (34895)

32 activit*.ti,ab. (20836)

33 interaction*.ti,ab. (6648)

34 (project or projects).ti,ab. (26695)

35 intervention*.ti,ab. (27280)

36 initiative*.ti,ab. (15221)

37 scheme.ti,ab. (7787)

38 visit*.ti,ab. (5684)

39 reading.ti,ab. (1762)

40 (play or playing or playtime).ti,ab. (7707)

41 music.ti,ab. (944)

42 boardgame*.ti,ab. (0)

43 games.ti,ab. (790)

44 voluntary.ti,ab. (10402)

45 volunteering.ti,ab. (1431)

46 mentor*.ti,ab. (1383)

47 or/31-46 (125313)

48 care home*.ti,ab. (5287)

49 nursing home*.ti,ab. (4646)

50 residential care.ti,ab. (5649)

51 ((senior or elderly or old) adj day care).ti,ab. (7)

52 ((hospital* or ward) adj3 geriatric*).ti,ab. (236)

53 community.ti,ab. (49136)

54 (sheltered adj (housing or accommodation)).ti,ab. (1468)

55 (retirement adj (home* or village* or complex*)).ti,ab. (182)

56 (abbeyfield or almshouse*).ti,ab. (113)

57 (geriatric adj (institution* or care)).ti,ab. (167)

58 assisted living.ti,ab. (461)

59 own home.ti,ab. (455)

60 (preschool or preschools).ti,ab. (446)

61 playgroup*.ti,ab. (97)

62 (school or schools or college*).ti,ab. (24710)

63 (nursery or nurseries).ti,ab. (690)

64 kindergarten*.ti,ab. (93)

65 play setting*.ti,ab. (7)

66 (child care setting* or childcare setting*).ti,ab. (112)

67 (child* adj2 day care).ti,ab. (155)

68 or/48-67 (84371)

69 7 and 16 and 30 and 47 (325)

70 7 and 16 and 47 and 68 (259)

71 7 and 30 and 47 and 68 (285)

72 6 or 69 or 70 or 71 (538)

Database: HMIC Health Management Information Consortium <1979 to May 2021>

Search Strategy:

--------------------------------------------------------------------------------

1 (intergenerational or inter generational).ti,ab. (182)

2 cross age.ti,ab. (0)

3 across generation*.ti,ab. (37)

4 cross generation*.ti,ab. (11)

5 ((generations or different age groups or all ages or all age groups or mixed ages or mixed age groups or (old* adj2 young*)) adj5 (together or social engagement or connecting)).ti,ab. (10)

6 (intergenerational adj2 (program* or intervention*)).ti. (2)

7 or/1-5 (223)

8 child*.ti,ab. (31008)

9 (young adj (person or people or male* or female*)).ti,ab. (5636)

10 (youth* or teen*).ti,ab. (3627)

11 young offender*.ti,ab. (338)

12 (school and pupil*).ti,ab. (380)

13 preschooler*.ti,ab. (50)

14 student*.ti,ab. (6683)

15 (girl or girls or boy or boys).ti,ab. (1776)

16 or/8-15 (42746)

17 dementia.ti,ab. (3002)

18 alzheimer*.ti,ab. (601)

19 old*.ti,ab. (18096)

20 elderly.ti,ab. (8811)

21 geriatric.ti,ab. (1288)

22 (residents or resident).ti,ab. (4929)

23 (elder or elders).ti,ab. (564)

24 (retired or retiree*).ti,ab. (314)

25 veteran*.ti,ab. (469)

26 grandfriend*.ti,ab. (0)

27 seniors.ti,ab. (156)

28 (senior adj citizen*).ti,ab. (49)

29 (centarian* or centenarian* or nonagenarian* or octagenarian* or octogenarian* or sexagenarian* or septuagenarian*).ti,ab. (28)

30 or/17-29 (32364)

31 program*.ti,ab. (28482)

32 activit*.ti,ab. (15956)

33 interaction*.ti,ab. (4491)

34 (project or projects).ti,ab. (18310)

35 intervention*.ti,ab. (19407)

36 initiative*.ti,ab. (10545)

37 scheme.ti,ab. (6845)

38 visit*.ti,ab. (10874)

39 reading.ti,ab. (1249)

40 (play or playing or playtime).ti,ab. (4805)

41 music.ti,ab. (240)

42 boardgame*.ti,ab. (0)

43 games.ti,ab. (271)

44 voluntary.ti,ab. (6203)

45 volunteering.ti,ab. (279)

46 mentor*.ti,ab. (705)

47 or/31-46 (99116)

48 care home*.ti,ab. (1834)

49 nursing home*.ti,ab. (2158)

50 residential care.ti,ab. (2343)

51 ((senior or elderly or old) adj day care).ti,ab. (2)

52 ((hospital* or ward) adj3 geriatric*).ti,ab. (259)

53 community.ti,ab. (34580)

54 (sheltered adj (housing or accommodation)).ti,ab. (329)

55 (retirement adj (home* or village* or complex*)).ti,ab. (31)

56 (abbeyfield or almshouse*).ti,ab. (16)

57 (geriatric adj (institution* or care)).ti,ab. (99)

58 assisted living.ti,ab. (66)

59 own home.ti,ab. (187)

60 (preschool or preschools).ti,ab. (301)

61 playgroup*.ti,ab. (79)

62 (school or schools or college*).ti,ab. (12524)

63 (nursery or nurseries).ti,ab. (374)

64 kindergarten*.ti,ab. (59)

65 play setting*.ti,ab. (3)

66 (child care setting* or childcare setting*).ti,ab. (14)

67 (child* adj2 day care).ti,ab. (88)

68 or/48-67 (51037)

69 7 and 16 and 30 and 47 (8)

70 7 and 16 and 47 and 68 (9)

71 7 and 30 and 47 and 68 (6)

72 6 or 69 or 70 or 71 (18)

CINAHL via EBSCOhost

S135 S66 OR S132 OR S133 OR S134

S134 S67 and S92 and S109 and S131

S133 S67 and S77 and S109 and S131

S132 S67 AND S77 AND S92 AND S109

S131 S110 OR S111 OR S112 OR S113 OR S114 OR S115 OR S116 OR S117 OR S118 OR S119 OR S120 OR S121 OR S122 OR S123 OR S124 OR S125 OR S126 OR S127 OR S128 OR S129 OR S130

S130 TI ( (abbeyfield or almshouse*)) OR AB ( (abbeyfield or almshouse*))

S129 TI (child* N2 day care) OR AB (child* N2 day care)

S128 TI ( (child care setting* or childcare setting*)) OR AB ( (child care setting* or childcare setting*))

S127 TI play setting* OR AB play setting*

S126 TI kindergarten* OR AB kindergarten*

S125 TI ( (nursery or nurseries)) OR AB ( (nursery or nurseries))

S124 TI ( (school or schools or college*)) OR AB ( (school or schools or college*))

S123 TI playgroup* OR AB playgroup*

S122 TI ( (preschool or preschools)) OR AB ( (preschool or preschools))

S121 TI own home OR AB own home

S120 TI assisted living OR AB assisted living

S119 TI ( (geriatric N0 (institution* or care))) OR AB ( (geriatric N0 (institution* or care)))

S118 TI ( (retirement N0 (home* or village* or complex*))) OR AB ( (retirement N0 (home* or village* or complex*)))

S117 TI ( (sheltered N0 (housing or accommodation))) OR AB ( (sheltered N0 (housing or accommodation)))

S116 TI community OR AB community

S115 TI ( ((hospital* or ward) N3 geriatric*)) OR AB ( ((hospital* or ward) N3 geriatric*))

S114 TI ( ((senior or elderly or old) N0 day care)) OR AB ( ((senior or elderly or old) N0 day care))

S113 TI residential care OR AB residential care

S112 TI nursing home* OR AB nursing home*

S111 TI care home* OR AB care home*

S110 (MH “Nursing Homes”)

S109 S93 OR S94 OR S95 OR S96 OR S97 OR S98 OR S99 OR S100 OR S101 OR S102 OR S103 OR S104 OR S105 OR S106 OR S107 OR S108

S108 TI mentor* OR AB mentor*

S107 TI volunteering OR AB volunteering

S106 TI voluntary OR AB voluntary

S105 TI games OR AB games

S104 TI boardgame* OR AB boardgame*

S103 TI music OR AB music

S102 TI ( (play or playing or playtime)) OR AB ( (play or playing or playtime))

S101 TI reading OR AB reading

S100 TI visit* OR AB visit*

S99 TI scheme OR AB scheme

S98 TI initiative* OR AB initiative*

S97 TI intervention* OR AB intervention*

S96 TI ( (project or projects)) OR AB ( (project or projects))

S95 TI interaction* OR AB interaction*

Database - CINAHL Display

S94 TI activit* OR AB activit*

S93 TI program* OR AB program*

S92 S78 OR S79 OR S80 OR S81 OR S82 OR S83 OR S84 OR S85 OR S86 OR S87 OR S88 OR S89 OR S90 OR S91

S91 TI ( (centarian* or centenarian* or nonagenarian* or octagenarian* or octogenarian* or sexagenarian* or septuagenarian*)) AND AB ( (centarian* or centenarian* or nonagenarian* or octagenarian* or octogenarian* or sexagenarian* or septuagenarian*))

S90 TI (senior N0 citizen*) OR AB (senior N0 citizen*)

S89 TI seniors OR AB seniors

S88 TI grandfriend* OR AB grandfriend*

S87 TI veteran* OR AB veteran*

S86 TI ( (retired or retiree*)) OR AB ( (retired or retiree*))

S85 TI ( (elder or elders)) OR AB ( (elder or elders))

S84 TI ( (residents or resident)) OR AB ( (residents or resident))

S83 TI geriatric OR AB geriatric

S82 TI elderly OR AB elderly

S81 TI old* OR AB old*

S80 TI alzheimer* OR AB alzheimer*

S79 TI dementia OR AB dementia

S78 (MH “Aged”)

S77 S68 OR S69 OR S70 OR S71 OR S72 OR S73 OR S74 OR S75 OR S76

S76 TI ( (girl or girls or boy or boys)) OR AB ( (girl or girls or boy or boys))

S75 TI student* OR AB student*

S74 TI preschooler* OR AB preschooler*

S73 TI ( (school and pupil*)) OR AB ( (school and pupil*))

S72 TI young offender* OR AB young offender*

S71 TI ( (youth* or teen*)) OR AB ( (youth* or teen*))

S70 TI ( (young N0 (person or people or male* or female*))) OR AB ( (young N0 (person or people or male* or female*)))

S69 TI child* OR AB child*

S68 (MH “Adolescence”) OR (MH “Child”) OR (MH “Child, Preschool”)

S67 S60 OR S61 OR S62 OR S63 OR S64 OR S65

S66 TI (intergenerational N2 (program* or intervention*))

S65 TI ( ((generations or different age groups or all ages or all age groups or mixed ages or mixed age groups or (old* adj2 young*)) N5 (together or social engagement or connecting))) OR AB ( ((generations or different age groups or all ages or all age groups or mixed ages or mixed age groups or (old* adj2 young*)) N5 (together or social engagement or connecting)))

S64 TI cross generation* OR AB cross generation*

S63 TI across generation* OR AB across generation*

S62 TI cross age OR AB cross age

S61 TI ( (intergenerational or inter generational)) OR AB ( (intergenerational or inter generational))

S60 (MH “Intergenerational Relations”)

S59 TI ( (retirement N0 (home* or village* or complex*))) OR AB ( (retirement N0 (home* or village* or complex*)))

S58 TI ( (sheltered N0 (housing or accommodation))) OR AB ( (sheltered N0 (housing or accommodation)))

S57 TI community OR AB community

S56 TI ( ((hospital* or ward) N3 geriatric*)) OR AB ( ((hospital* or ward) N3 geriatric*))

S55 TI ( ((senior or elderly or old) N0 day care)) OR AB ( ((senior or elderly or old) N0 day care))

S54 TI residential care OR AB residential care

S53 TI nursing home* OR AB nursing home*

S52 TI care home* OR AB care home*

S51 (MH “Nursing Homes”)

S50 S34 OR S35 OR S36 OR S37 OR S38 OR S39 OR S40 OR S41 OR S42 OR S43 OR S44 OR S45 OR S46 OR S47 OR S48 OR S49

S49 TI mentor* OR AB mentor*

S48 TI volunteering OR AB volunteering

S47 TI voluntary OR AB voluntary

S46 TI games OR AB games

S45 TI boardgame* OR AB boardgame*

S44 TI music OR AB music

S43 TI ( (play or playing or playtime)) OR AB ( (play or playing or playtime))

S42 TI reading OR AB reading

S41 TI visit* OR AB visit*

S40 TI scheme OR AB scheme

S39 TI initiative* OR AB initiative*

S38 TI intervention* OR AB intervention*

S37 TI ( (project or projects)) OR AB ( (project or projects))

S36 TI interaction* OR AB interaction*

S35 TI activit* OR AB activit*

S34 TI program* OR AB program*

Database - CINAHL Display

S33 S19 OR S20 OR S21 OR S22 OR S23 OR S24 OR S25 OR S26 OR S27 OR S28 OR S29 OR S30 OR S31 OR S32

S32 TI ( (centarian* or centenarian* or nonagenarian* or octagenarian* or octogenarian* or sexagenarian* or septuagenarian*)) AND AB ( (centarian* or centenarian* or nonagenarian* or octagenarian* or octogenarian* or sexagenarian* or septuagenarian*))

S31 TI (senior N0 citizen*) OR AB (senior N0 citizen*)

S30 TI seniors OR AB seniors

S29 TI grandfriend* OR AB grandfriend*

S28 TI veteran* OR AB veteran*

S27 TI ( (retired or retiree*)) OR AB ( (retired or retiree*))

S26 TI ( (elder or elders)) OR AB ( (elder or elders))

S25 TI ( (residents or resident)) OR AB ( (residents or resident))

S24 TI geriatric OR AB geriatric

S23 TI elderly OR AB elderly

S22 TI old* OR AB old*

S21 TI alzheimer* OR AB alzheimer*

S20 TI dementia OR AB dementia

S19 (MH “Aged”)

S18 S9 OR S10 OR S11 OR S12 OR S13 OR S14 OR S15 OR S16 OR S17

S17 TI ( (girl or girls or boy or boys)) OR AB ( (girl or girls or boy or boys))

S16 TI student* OR AB student*

S15 TI preschooler* OR AB preschooler*

S14 TI ( (school and pupil*)) OR AB ( (school and pupil*))

S13 TI young offender* OR AB young offender*

S12 TI ( (youth* or teen*)) OR AB ( (youth* or teen*))

S11 TI ( (young N0 (person or people or male* or female*))) OR AB ( (young N0 (person or people or male* or female*)))

S10 TI child* OR AB child*

S9 (MH “Adolescence”) OR (MH “Child”) OR (MH “Child, Preschool”)

S8 S1 OR S2 OR S3 OR S4 OR S5 OR S6

S7 TI (intergenerational N2 (program* or intervention*))

S6 TI ( ((generations or different age groups or all ages or all age groups or mixed ages or mixed age groups or (old* adj2 young*)) N5 (together or social engagement or connecting))) OR AB ( ((generations or different age groups or all ages or all age groups or mixed ages or mixed age groups or (old* adj2 young*)) N5 (together or social engagement or connecting)))

S5 TI cross generation* OR AB cross generation*

S4 TI across generation* OR AB across generation*

S3 TI cross age OR AB cross age

S2 TI ( (intergenerational or inter generational)) OR AB ( (intergenerational or inter generational))

S1 (MH “Intergenerational Relations”)

AgeLine via EBSCOhost

S5 S3 OR S4

S4 TI ( (volunteer* or “voluntary”) W5 (school* or playgroup* or “play setting*“ or kindergarten*

or nurser*)) OR AB ( (volunteer* or “voluntary”) W5 (school* or playgroup* or “play setting*“ or kindergarten* or nurser*))

S3 S1 OR S2

S2 TI ( intergenerational W1 (program* or intervention* or project* or initiative* or scheme*)) OR AB ( intergenerational W1 (program* or intervention* or project* or initiative* or scheme*))

S1 DE “Intergenerational Programs”

ERIC via EBSCOhost

Search Terms Search Options

S4 S1 OR S2 OR S3

S3 TI intergenerational

S2 AB ( TI ( (volunteer* or “voluntary”) W5 (“old aged” or elderly or “geriatric” or pensioner* or veteran* or older))) OR TI ( TI ( (volunteer* or “voluntary”) W5 (“old aged” or elderly or “geriatric” or pensioner* or veteran* or older)))

S1 TI ( intergenerational W1 (program* or intervention* or project* or initiative* or scheme*)) OR AB ( intergenerational W1 (program* or intervention* or project* or initiative* or scheme*))

ASSIA via ProQuest

(intergenerational NEAR/2 (program* OR intervention*)) OR ((MAINSUBJECT.EXACT(“Intergenerational relationships”) OR (ti((intergenerational OR inter generational)) OR ab((intergenerational OR inter generational))) OR (ti(cross age) OR ab(cross age)) OR (ti(across generation*) OR ab(across generation*)) OR (ti(cross generation*) OR ab(cross generation*)) OR (((ti((generations OR different age groups OR all ages OR all age groups OR mixed ages OR mixed age groups)) OR ab((generations OR different age groups OR all ages OR all age groups OR mixed ages OR mixed age groups))) OR (ti((“old* and young*“ OR “old* people and young* people” OR “old* persons and young* persons” OR “old* generation* and young* generation*“)) OR ab((“old* and young*“ OR “old* people and young* people” OR “old* persons and young* persons” OR “old* generation* and young* generation*“)))) AND (ti((together OR social engagement OR connecting)) OR ab((together OR social engagement OR connecting))))) AND ((ti(child*) OR ab(child*)) OR (ti((young NEAR/0 (person OR people OR male* OR female*))) OR ab((young NEAR/0 (person OR people OR male* OR female*)))) OR

(ti((youth* OR teen*)) OR ab((youth* OR teen*))) OR (ti(young offender*) OR ab(young offender*)) OR (ti((school AND pupil*)) OR ab((school AND pupil*))) OR (ti(preschooler*) OR ab(preschooler*)) OR (ti(student*) OR ab(student*)) OR (ti((girl OR girls OR boy OR boys)) OR ab((girl OR girls OR boy OR boys)))) AND (MAINSUBJECT.EXACT(“Elderly people”) OR (ti(dementia) OR ab(dementia)) OR (ti(alzheimer*) OR ab(alzheimer*)) OR (ti(old*) OR ab(old*)) OR (ti(elderly) OR ab(elderly)) OR (ti(geriatric) OR ab(geriatric)) OR (ti((residents OR resident)) OR ab((residents OR resident))) OR (ti((elder OR elders)) OR ab((elder OR elders))) OR (ti((retired OR retiree*)) OR ab((retired OR retiree*))) OR (ti(veteran*) OR ab(veteran*)) OR (ti(grandfriend*) OR ab(grandfriend*)) OR grandfriend* OR (ti(seniors) OR ab(seniors)) OR (ti((senior NEAR/0 citizen*)) OR ab((senior NEAR/0 citizen*))) OR (ti((centarian* OR centenarian* OR nonagenarian* OR octagenarian* OR octogenarian* OR sexagenarian* OR septuagenarian*)) OR ab((centarian* OR centenarian* OR nonagenarian* OR octagenarian* OR octogenarian* OR sexagenarian* OR septuagenarian*)))) AND ((ti(intergeneration* NEAR/10 program*) OR ab(intergeneration* NEAR/10 program*)) OR (ti(activit*) OR ab(activit*)) OR (ti(interaction*) OR ab(interaction*)) OR (ti((intergeneration* NEAR/10 project OR intergeneration* NEAR/10 projects)) OR ab((intergeneration* NEAR/10 project OR intergeneration* NEAR/10 projects))) OR (ti(intergeneration* NEAR/10 intervention*) OR ab(intergeneration* NEAR/10 intervention*)) OR (ti(initiative*) OR ab(initiative*)) OR (ti(scheme) OR ab(scheme)) OR (ti(visit*) OR ab(visit*)) OR (ti(reading) OR ab(reading)) OR (ti((play OR playing OR playtime)) OR ab((play OR playing OR playtime))) OR (ti(music) OR ab(music)) OR (ti(boardgame*) OR ab(boardgame*)) OR (ti(games) OR ab(games)) OR (ti(volunteering) OR ab(volunteering)) OR (ti(mentor*) OR ab(mentor*)))) OR ((MAINSUBJECT.EXACT(“Intergenerational relationships”) OR (ti((intergenerational OR inter generational)) OR ab((intergenerational OR inter generational))) OR (ti(cross age) OR ab(cross age)) OR (ti(across generation*) OR ab(across generation*)) OR (ti(cross generation*) OR ab(cross generation*)) OR (((ti((generations OR different age groups OR all ages OR all age groups OR mixed ages OR mixed age groups)) OR ab((generations OR different age groups OR all ages OR all age groups OR mixed ages OR mixed age groups))) OR (ti((“old* and young*“ OR “old* people and young* people” OR “old* persons and young* persons” OR “old* generation* and young* generation*“)) OR ab((“old* and young*“ OR “old* people and young* people” OR “old* persons and young* persons” OR “old* generation* and young* generation*“)))) AND (ti((together OR social engagement OR connecting)) OR ab((together OR social engagement OR connecting))))) AND ((ti(child*) OR ab(child*)) OR (ti((young NEAR/0 (person OR people OR male* OR female*))) OR ab((young NEAR/0 (person OR people OR male* OR female*)))) OR (ti((youth* OR teen*)) OR ab((youth* OR teen*))) OR (ti(young offender*) OR ab(young offender*)) OR (ti((school AND pupil*)) OR ab((school AND pupil*))) OR (ti(preschooler*) OR ab(preschooler*)) OR (ti(student*) OR ab(student*)) OR (ti((girl OR girls OR boy OR boys)) OR ab((girl OR girls OR boy OR boys)))) AND ((ti(intergeneration* NEAR/10 program*) OR

ab(intergeneration* NEAR/10 program*)) OR (ti(activit*) OR ab(activit*)) OR (ti(interaction*) OR ab(interaction*)) OR (ti((intergeneration* NEAR/10 project OR intergeneration* NEAR/10 projects)) OR ab((intergeneration* NEAR/10 project OR intergeneration* NEAR/10 projects))) OR (ti(intergeneration* NEAR/10 intervention*) OR ab(intergeneration* NEAR/10 intervention*)) OR (ti(initiative*) OR ab(initiative*)) OR (ti(scheme) OR ab(scheme)) OR (ti(visit*) OR ab(visit*)) OR (ti(reading) OR ab(reading)) OR (ti((play OR playing OR playtime)) OR ab((play OR playing OR playtime))) OR (ti(music) OR ab(music)) OR (ti(boardgame*) OR ab(boardgame*)) OR (ti(games) OR ab(games)) OR (ti(volunteering) OR ab(volunteering)) OR (ti(mentor*) OR ab(mentor*))) AND ((MAINSUBJECT.EXACT(“Nursing homes”) OR MAINSUBJECT.EXACT(“Private nursing homes”)) OR (ti(care home*) OR ab(care home*)) OR (ti(nursing home*) OR ab(nursing home*)) OR (ti(residential care) OR ab(residential care)) OR (ti(((senior OR elderly OR old) NEAR/0 day care)) OR ab(((senior OR elderly OR old) NEAR/0 day care))) OR (ti(((hospital* OR ward) NEAR/3 geriatric*)) OR ab(((hospital* OR ward) NEAR/3 geriatric*))) OR (ti(community) OR ab(community)) OR (ti((sheltered NEAR/0 (housing OR accommodation))) OR ab((sheltered NEAR/0 (housing OR accommodation)))) OR (ti((retirement adj (home* OR village* OR complex*))) OR ab((retirement adj (home* OR village* OR complex*)))) OR (ti((retirement NEAR/0 (home* OR village* OR complex*))) OR ab((retirement NEAR/0 (home* OR village* OR complex*)))) OR (ti((abbeyfield OR almshouse*)) OR ab((abbeyfield OR almshouse*))) OR (ti((geriatric NEAR/0 (institution* OR care))) OR ab((geriatric NEAR/0 (institution* OR care)))) OR (ti(assisted living) OR ab(assisted living)) OR (ti(own home) OR ab(own home)) OR (ti((preschool OR preschools)) OR ab((preschool OR preschools))) OR (ti(playgroup*) OR ab(playgroup*)) OR (ti((school OR schools OR college*)) OR ab((school OR schools OR college*))) OR (ti((nursery OR nurseries)) OR ab((nursery OR nurseries))) OR (ti(kindergarten*) OR ab(kindergarten*)) OR (ti(play setting*) OR ab(play setting*)) OR (ti((child care setting* OR childcare setting*)) OR ab((child care setting* OR childcare setting*))) OR (ti((child* NEAR/2 day care)) OR ab((child* NEAR/2 day care))))) OR ((MAINSUBJECT.EXACT(“Intergenerational relationships”) OR (ti((intergenerational OR inter generational)) OR ab((intergenerational OR inter generational))) OR (ti(cross age) OR ab(cross age)) OR (ti(across generation*) OR ab(across generation*)) OR (ti(cross generation*) OR ab(cross generation*)) OR (((ti((generations OR different age groups OR all ages OR all age groups OR mixed ages OR mixed age groups)) OR ab((generations OR different age groups OR all ages OR all age groups OR mixed ages OR mixed age groups))) OR (ti((“old* and young*“ OR “old* people and young* people” OR “old* persons and young* persons” OR “old* generation* and young* generation*“)) OR ab((“old* and young*“ OR “old* people and young* people” OR “old* persons and young* persons” OR “old* generation* and young* generation*“)))) AND (ti((together OR social engagement OR connecting)) OR ab((together OR social engagement OR connecting))))) AND (MAINSUBJECT.EXACT(“Elderly people”) OR (ti(dementia) OR ab(dementia)) OR (ti(alzheimer*) OR ab(alzheimer*)) OR (ti(old*) OR ab(old*))

OR (ti(elderly) OR ab(elderly)) OR (ti(geriatric) OR ab(geriatric)) OR (ti((residents OR resident)) OR ab((residents OR resident))) OR (ti((elder OR elders)) OR ab((elder OR elders))) OR (ti((retired OR retiree*)) OR ab((retired OR retiree*))) OR (ti(veteran*) OR ab(veteran*)) OR (ti(grandfriend*) OR ab(grandfriend*)) OR grandfriend* OR (ti(seniors) OR ab(seniors)) OR (ti((senior NEAR/0 citizen*)) OR ab((senior NEAR/0 citizen*))) OR (ti((centarian* OR centenarian* OR nonagenarian* OR octagenarian* OR octogenarian* OR sexagenarian* OR septuagenarian*)) OR ab((centarian* OR centenarian* OR nonagenarian* OR octagenarian* OR octogenarian* OR sexagenarian* OR septuagenarian*)))) AND ((ti(intergeneration* NEAR/10 program*) OR ab(intergeneration* NEAR/10 program*)) OR (ti(activit*) OR ab(activit*)) OR (ti(interaction*) OR ab(interaction*)) OR (ti((intergeneration* NEAR/10 project OR intergeneration* NEAR/10 projects)) OR ab((intergeneration* NEAR/10 project OR intergeneration* NEAR/10 projects))) OR (ti(intergeneration* NEAR/10 intervention*) OR ab(intergeneration* NEAR/10 intervention*)) OR (ti(initiative*) OR ab(initiative*)) OR (ti(scheme) OR ab(scheme)) OR (ti(visit*) OR ab(visit*)) OR (ti(reading) OR ab(reading)) OR (ti((play OR playing OR playtime)) OR ab((play OR playing OR playtime))) OR (ti(music) OR ab(music)) OR (ti(boardgame*) OR ab(boardgame*)) OR (ti(games) OR ab(games)) OR (ti(volunteering) OR ab(volunteering)) OR (ti(mentor*) OR ab(mentor*))) AND ((MAINSUBJECT.EXACT(“Nursing homes”) OR MAINSUBJECT.EXACT(“Private nursing homes”)) OR (ti(care home*) OR ab(care home*)) OR (ti(nursing home*) OR ab(nursing home*)) OR (ti(residential care) OR ab(residential care)) OR (ti(((senior OR elderly OR old) NEAR/0 day care)) OR ab(((senior OR elderly OR old) NEAR/0 day care))) OR (ti(((hospital* OR ward) NEAR/3 geriatric*)) OR ab(((hospital* OR ward) NEAR/3 geriatric*))) OR (ti(community) OR ab(community)) OR (ti((sheltered NEAR/0 (housing OR accommodation))) OR ab((sheltered NEAR/0 (housing OR accommodation)))) OR (ti((retirement adj (home* OR village* OR complex*))) OR ab((retirement adj (home* OR village* OR complex*)))) OR (ti((retirement NEAR/0 (home* OR village* OR complex*))) OR ab((retirement NEAR/0 (home* OR village* OR complex*)))) OR (ti((abbeyfield OR almshouse*)) OR ab((abbeyfield OR almshouse*))) OR (ti((geriatric NEAR/0 (institution* OR care))) OR ab((geriatric NEAR/0 (institution* OR care)))) OR (ti(assisted living) OR ab(assisted living)) OR (ti(own home) OR ab(own home)) OR (ti((preschool OR preschools)) OR ab((preschool OR preschools))) OR (ti(playgroup*) OR ab(playgroup*)) OR (ti((school OR schools OR college*)) OR ab((school OR schools OR college*))) OR (ti((nursery OR nurseries)) OR ab((nursery OR nurseries))) OR (ti(kindergarten*) OR ab(kindergarten*)) OR (ti(play setting*) OR ab(play setting*)) OR (ti((child care setting* OR childcare setting*)) OR ab((child care setting* OR childcare setting*))) OR (ti((child* NEAR/2 day care)) OR ab((child* NEAR/2 day care)))))

ProQuest Dissertations and Theses

ti((intergenerational NEAR/2 (program* OR intervention*))) OR ((ti((intergenerational OR inter generational)) OR ti(cross age) OR ti(cross generation*) OR ti((generations OR different age groups OR all ages OR all age groups OR mixed ages OR mixed age groups))) AND ((ti(child*) OR ab(child*)) OR (ti((young NEAR/0 (person OR people OR male* OR female*))) OR ab((young NEAR/0 (person OR people OR male* OR female*)))) OR (ti((youth* OR teen*)) OR ab((youth* OR teen*))) OR (ti(young offender*) OR ab(young offender*)) OR (ti((school AND pupil*)) OR ab((school AND pupil*))) OR (ti(preschooler*) OR ab(preschooler*)) OR (ti(student*) OR ab(student*)) OR (ti((girl OR girls OR boy OR boys)) OR ab((girl OR girls OR boy OR boys)))) AND ((ti(dementia) OR ab(dementia)) OR (ti(alzheimer) OR ab(alzheimer)) OR (ti(old*) OR ab(old*)) OR (ti(elderly) OR ab(elderly)) OR (ti(geriatric) OR ab(geriatric)) OR (ti((residents OR resident)) OR ab((residents OR resident))) OR (ti((elder OR elders)) OR ab((elder OR elders))) OR (ti((retired OR retiree*)) OR ab((retired OR retiree*))) OR (ti(veteran*) OR ab(veteran*)) OR (ti(grandfriend*) OR ab(grandfriend*)) OR (ti(seniors) OR ab(seniors)) OR (ti((senior NEAR/0 citizen*)) OR ab((senior NEAR/0 citizen*))) OR (ti((centarian* OR centenarian* OR nonagenarian* OR octagenarian* OR octogenarian* OR sexagenarian* OR septuagenarian*)) OR ab((centarian* OR centenarian* OR nonagenarian* OR octagenarian* OR octogenarian* OR sexagenarian* OR septuagenarian*)))) AND ((ti(intergenerational NEAR/10 program*) OR ab(intergenerational NEAR/10 program*)) OR (ti(activit*) OR ab(activit*)) OR (ti(interaction*) OR ab(interaction*)) OR (ti(intergenerational NEAR/10 (project OR projects)) OR ab(intergenerational NEAR/10 (project OR projects))) OR (ti(intergenerational NEAR/10 intervention*) OR ab(intergenerational NEAR/10 intervention*)) OR (ti(initiative*) OR ab(initiative*)) OR (ti(scheme) OR ab(scheme)) OR (ti(visit*) OR ab(visit*)) OR (ti(reading) OR ab(reading)) OR (ti((play OR playing OR playtime)) OR ab((play OR playing OR playtime))) OR (ti(music) OR ab(music)) OR (ti(boardgame*) OR ab(boardgame*)) OR (ti(games) OR ab(games)) OR (ti(voluntary) OR ab(voluntary)) OR (ti(volunteering) OR ab(volunteering)) OR (ti(mentor*) OR ab(mentor*)))) OR ((ti((intergenerational OR inter generational)) OR ti(cross age) OR ti(cross generation*) OR ti((generations OR different age groups OR all ages OR all age groups OR mixed ages OR mixed age groups))) AND ((ti(child*) OR ab(child*)) OR (ti((young NEAR/0 (person OR people OR male* OR female*))) OR ab((young NEAR/0 (person OR people OR male* OR female*)))) OR (ti((youth* OR teen*)) OR ab((youth* OR teen*))) OR (ti(young offender*) OR ab(young offender*)) OR (ti((school AND pupil*)) OR ab((school AND pupil*))) OR (ti(preschooler*) OR ab(preschooler*)) OR (ti(student*) OR ab(student*)) OR (ti((girl OR girls OR boy OR boys)) OR ab((girl OR girls OR boy OR boys)))) AND ((ti(intergenerational NEAR/10 program*) OR ab(intergenerational NEAR/10 program*)) OR (ti(activit*) OR ab(activit*)) OR (ti(interaction*) OR ab(interaction*)) OR (ti(intergenerational NEAR/10 (project OR projects)) OR ab(intergenerational NEAR/10 (project OR projects))) OR (ti(intergenerational NEAR/10 intervention*) OR ab(intergenerational NEAR/10 intervention*)) OR (ti(initiative*) OR ab(initiative*)) OR (ti(scheme) OR ab(scheme)) OR (ti(visit*)

OR ab(visit*)) OR (ti(reading) OR ab(reading)) OR (ti((play OR playing OR playtime)) OR ab((play OR playing OR playtime))) OR (ti(music) OR ab(music)) OR (ti(boardgame*) OR ab(boardgame*)) OR (ti(games) OR ab(games)) OR (ti(voluntary) OR ab(voluntary)) OR (ti(volunteering) OR ab(volunteering)) OR (ti(mentor*) OR ab(mentor*))) AND ((ti(care home*) OR ab(care home*)) OR (ti(nursing home*) OR ab(nursing home*)) OR (ti(residential care) OR ab(residential care)) OR (ti(((senior OR elderly OR old) NEAR/0 day care)) OR ab(((senior OR elderly OR old) NEAR/0 day care))) OR (ti(((hospital* OR ward) NEAR/3 geriatric*)) OR ab(((hospital* OR ward) NEAR/3 geriatric*))) OR (ti(community) OR ab(community)) OR (ti((sheltered NEAR/0 (housing OR accommodation))) OR ab((sheltered NEAR/0 (housing OR accommodation)))) OR (ti((retirement NEAR/0 (home* OR village* OR complex*))) OR ab((retirement NEAR/0 (home* OR village* OR complex*)))) OR (ti((abbeyfield OR almshouse*)) OR ab((abbeyfield OR almshouse*))) OR (ti((geriatric NEAR/0 (institution* OR care))) OR ab((geriatric NEAR/0 (institution* OR care)))) OR (ti(assisted living) OR ab(assisted living)) OR (ti(own home) OR ab(own home)) OR (ti((preschool OR preschools)) OR ab((preschool OR preschools))) OR (ti(playgroup*) OR ab(playgroup*)) OR (ti((school OR schools OR college*)) OR ab((school OR schools OR college*))) OR (ti((nursery OR nurseries)) OR ab((nursery OR nurseries))) OR (ti(kindergarten*) OR ab(kindergarten*)) OR (ti(play setting*) OR ab(play setting*)) OR (ti((child* NEAR/2 day care)) OR ab((child* NEAR/2 day care))))) OR ((ti((intergenerational OR inter generational)) OR ti(cross age) OR ti(cross generation*) OR ti((generations OR different age groups OR all ages OR all age groups OR mixed ages OR mixed age groups))) AND ((ti(dementia) OR ab(dementia)) OR (ti(alzheimer) OR ab(alzheimer)) OR (ti(old*) OR ab(old*)) OR (ti(elderly) OR ab(elderly)) OR (ti(geriatric) OR ab(geriatric)) OR (ti((residents OR resident)) OR ab((residents OR resident))) OR (ti((elder OR elders)) OR ab((elder OR elders))) OR (ti((retired OR retiree*)) OR ab((retired OR retiree*))) OR (ti(veteran*) OR ab(veteran*)) OR (ti(grandfriend*) OR ab(grandfriend*)) OR (ti(seniors) OR ab(seniors)) OR (ti((senior NEAR/0 citizen*)) OR ab((senior NEAR/0 citizen*))) OR (ti((centarian* OR centenarian* OR nonagenarian* OR octagenarian* OR octogenarian* OR sexagenarian* OR septuagenarian*)) OR ab((centarian* OR centenarian* OR nonagenarian* OR octagenarian* OR octogenarian* OR sexagenarian* OR septuagenarian*)))) AND ((ti(intergenerational NEAR/10 program*) OR ab(intergenerational NEAR/10 program*)) OR (ti(activit*) OR ab(activit*)) OR (ti(interaction*) OR ab(interaction*)) OR (ti(intergenerational NEAR/10 (project OR projects)) OR ab(intergenerational NEAR/10 (project OR projects))) OR (ti(intergenerational NEAR/10 intervention*) OR ab(intergenerational NEAR/10 intervention*)) OR (ti(initiative*) OR ab(initiative*)) OR (ti(scheme) OR ab(scheme)) OR (ti(visit*) OR ab(visit*)) OR (ti(reading) OR ab(reading)) OR (ti((play OR playing OR playtime)) OR ab((play OR playing OR playtime))) OR (ti(music) OR ab(music)) OR (ti(boardgame*) OR ab(boardgame*)) OR (ti(games) OR ab(games)) OR (ti(voluntary) OR ab(voluntary)) OR (ti(volunteering) OR ab(volunteering)) OR (ti(mentor*) OR ab(mentor*))) AND

((ti(care home*) OR ab(care home*)) OR (ti(nursing home*) OR ab(nursing home*)) OR (ti(residential care) OR ab(residential care)) OR (ti(((senior OR elderly OR old) NEAR/0 day care)) OR ab(((senior OR elderly OR old) NEAR/0 day care))) OR (ti(((hospital* OR ward) NEAR/3 geriatric*)) OR ab(((hospital* OR ward) NEAR/3 geriatric*))) OR (ti(community) OR ab(community)) OR (ti((sheltered NEAR/0 (housing OR accommodation))) OR ab((sheltered NEAR/0 (housing OR accommodation)))) OR (ti((retirement NEAR/0 (home* OR village* OR complex*))) OR ab((retirement NEAR/0 (home* OR village* OR complex*)))) OR (ti((abbeyfield OR almshouse*)) OR ab((abbeyfield OR almshouse*))) OR (ti((geriatric NEAR/0 (institution* OR care))) OR ab((geriatric NEAR/0 (institution* OR care)))) OR (ti(assisted living) OR ab(assisted living)) OR (ti(own home) OR ab(own home)) OR (ti((preschool OR preschools)) OR ab((preschool OR preschools))) OR (ti(playgroup*) OR ab(playgroup*)) OR (ti((school OR schools OR college*)) OR ab((school OR schools OR college*))) OR (ti((nursery OR nurseries)) OR ab((nursery OR nurseries))) OR (ti(kindergarten*) OR ab(kindergarten*)) OR (ti(play setting*) OR ab(play setting*)) OR (ti((child* NEAR/2 day care)) OR ab((child* NEAR/2 day care)))))

SSCI and CPSI-S via Web of Science

## 2 Table of excluded studies

| Reason for Exclusion |  |
| --- | --- |
| Wrong study type/ descriptive piece | 1. Project for Academic Motivation: older adult volunteers in schools: Winnetka Public Schools, Winnetka, IL; 1969. 6p. p. |
|  | 2. Juneau seniors say yes to an intergenerational home chore service. Aging. 1983(340):34. |
|  | 3. Intergenerational programs: a synthesis of findings research and demonstration projects: National Association of State Units on Aging, Washington, DC; 1984. 39p. p. |
|  | 4. NCOA Family Friends volunteers enrich lives of handicapped children. Perspective on Aging. 1984;13(6):5-8. |
|  | 5. Project Get in SHAPE: Spelman Health Awareness Project for the Elderly: final report: Spelman College, Atlanta, GA; 1984. 183p. p. |
|  | 6. Volunteer youth visitation and accident prevention program for the isolated and frail elderly: Council of Jewish Organizations of Boro Park, Brooklyn, NY; 1984. 94p. p. |
|  | 7. Senior/Student Shared Housing Project: University of Utah, Intermountain West Long Term Care Gerontology Center, Salt Lake City, UT; 1987. 81p. p. |
|  | 8. Volunteer senior aides: the Pasadena family friends project: County of Los Angeles, Dept. of Community and Senior Citizens Services, Los Angeles, CA; 1994. 22p. p. |
|  | 9. Iowa program hopes to build intergenerational connections. Aging News Alert. 2003(16):5-6. |
|  | 10. Intergenerational. Cross-generational tutoring program benefits retirees, students alike. Aging News Alert. 2010:1p-p. |
|  | 11. Active Generations: Seniors Helping Kids Stay Healthy. School Health Alert. 2012;28(3):4-. |
|  | 12. City trials intergenerational program. Australian Journal of Dementia Care. 2018;7(2):5-. |
|  | 13. Introducing the Intergenerational Care Project. Australian Journal of Dementia Care. 2019;8(3):27-. |
|  | 14. Adams PL. Primary sources and senior citizens in the classroom. American Archivist. 1987;50(2):239-42. |
|  | 15. Allen M. Channel One: an intergenerational program at work. Children today. 1986;15(3):32-4. |
|  | 16. Amy G. The best of both worlds: a closer look at creating spaces that connect young and old. 2019:32. |
|  | 17. Anonymous. The reciprocity of student participation in compassionate community: An example of university social responsibility. Progress in Palliative Care. 2020;28(2):184. |
|  | 18. Bachmann IM. Aging in urban Japan – intergenerational reading in Tokyo. Working with Older People: Community Care Policy & Practice. 2014;18(1):24-9. |
|  | 19. Ball A, Cummerson R. More than words -- intergenerational participation and mental health. Mental Health & Social Inclusion. 2011;15(4):175-82. |
|  | 20. Banerjee N, Litow SS. Older and wiser: an intergenerational drop-out prevention effort: Interface, New York, NY; 1988. 10p. p. |
|  | 21. Becky R. Buds and Blossoms. Journal of Dementia Care. 2011;19(5):14-5. |
|  | 22. Bell T, et al., Tressler-Lutheran Service Association ICHPA. Mechanicsburg Area Phone Pal: An Intergenerational Telephone Reassurance Program for School-Age Children. How-to-Manual. 1987. |
|  | 23. Bennett K. Veterans in schools volunteer program. Australasian Journal on Ageing. 2000;19(1):66-. |
|  | 24. Berenbaum RLJ, Zweibach DF. Young adults with Down syndrome as caregivers for the elderly with dementia: an intergenerational project. Journal of Gerontological Social Work. 1996;26(3-4):159-70. |
|  | 25. Berkson J, Griggs SA. An intergenerational program at a middle school. School Counselor. 1986;34(2):140-3. |
|  | 26. Beynon CA, Little B, O’Regan N, McNaughton K, Beynon JG, Hutchison JMJL. Singing my way back to you: Learnings from the intergenerational choir project for singers with alzheimer’s disease, their caregivers, music educators, and students. Alzheimer’s and Dementia. 2016;12(7 Supplement):P799. |
|  | 27. Brady PE. Intergenerational child care: a venture into the future. Nursing Homes Long Term Care Management. 1997;46(4):20. |
|  | 28. Brandt-Saret B, Hubers CD, Mercure S. Latch-Key Phone Pals Program: final report: Elder Services of the Merrimack Valley, Inc., Lawrence, MA; 1993. 22p. p. |
|  | 29. Bressler J. The impact of intergenerational programs on long-term care residents. Gerontologist. 2001;41:307-. |
|  | 30. Brownell C. ART THERAPY AS A MEANS TO DECREASE PASSIVE BEHAVIORS IN PATIENTS WITH DEMENTIA: AN INTERGENERATIONAL ART PROGRAM. Gerontologist. 2008;48:639-. |
|  | 31. Burris HO. Design and Implementation of an Intergenerational Program That Fosters Creativity and Learning for Preschool Children. 1988. |
|  | 32. Butts DM, Chana K. Intergenerational programs promote active aging. Journal on Active Aging. 2007;6(4):34. |
|  | 33. Camareno N. An Analysis of Music Therapy and Music Education Intergenerational Programs and Their Importance to The Psychosocial, Physical, and Cognitive Needs of Older Adults [M.M.]. Ann Arbor: The Florida State University; 2019. |
|  | 34. Cambridge J, Simandiraki A. Interactive Intergenerational Learning in the Context of CAS in the IB Diploma Programme: A UK Case Study. Journal of Research in International Education. 2006;5(3):347-66. |
|  | 35. Camp CJ, Lee MM. Montessori-Based Activities as a Trans-Generational Interface for Persons with Dementia and Preschool Children. Journal of intergenerational relationships. 2011;9(4):366-73. |
|  | 36. Camp CJ, Orsulic-Jeras S, Lee MM, Judge KS. Effects of a Montessori-Based Intergenerational Program on Engagement and Affect for Adult Day Care Clients With Dementia. Successful aging through the life span: Intergenerational issues in health. 2005:159-76. |
|  | 37. Campanelli L, Leviton D. Intergenerational health promotion and rehabilitation: the Adult Health and Development Program model. Topics in Geriatric Rehabilitation. 1989;4(3):61-9. |
|  | 38. Carter-Tod D, Roussell JM. Implementing Intergenerational Electronic Communications into the Curriculum: Results of a Year-Long Case Study Featuring Second Graders and Senior Citizens. 1998. |
|  | 39. Chase CA. The effect of an intergenerational e -mail pen pal project on the attitudes of college students toward older adults [Ed.D.]. Ann Arbor: Ball State University; 2005. |
|  | 40. Chen SY. LEARNING TOGETHER ACROSS GENERATIONS: LEARNING IMPACT FROM A GERONTOLOGY INTERGENERATIONAL LEARNING CLASSROOM. Gerontologist. 2015;55:744-. |
|  | 41. Cherry DL, Benest FR, Gates B, White J. Intergenerational service programs: meeting shared needs of young and old. The Gerontologist. 1985;25(2):126-9. |
|  | 42. Ching-Ling L, Menders A, Jarrott S. Schools for all ages: how intergenerational programs nurture the youngest-young and oldest-old. Aging Today. 2011;32(5):9-12. |
|  | 43. Chonody J. Connecting older adults through multimedia: an intergenerational reminiscence program. Australasian Journal on Ageing. 2012;31:13-. |
|  | 44. Connolly MR, Jr. How Do You Spell Friendship? An Intergenerational Spelling Bee. Clearing House. 1993;66(5):267-8. |
|  | 45. Correia RH, Klea L, Campbell G, Costa AP. Fostering intergenerational education: An experiential learning program for medical students and older adults. Canadian medical education journal. 2020;11(5):e74-e7. |
|  | 46. Crites M, et al. The Agelink Project Replication Manual: An Intergenerational School-Age Child-Care Program. 1990. |
|  | 47. Cuevas R. I Can Help. Phi Delta Kappan. 2000;82(4):316. |
|  | 48. Cusicanqui M, Salmon R. Seniors, Small Fry, and Song: A Group Work Libretto of an Intergenerational Singing Group. Group work and aging: Issues in practice, research, and education. 2004:189-210. |
|  | 49. Danigelis NL, Fengler AP. No place like home: intergenerational homesharing through social exchange: Columbia University Press, New York, NY; 1991. 327p. p. |
|  | 50. David J, Yeung M, Vu J, Got T, Mackinnon C. Connecting the young and the young at heart: An intergenerational music program. Journal of Intergenerational Relationships. 2018;16(3):330-8. |
|  | 51. Davis K. Helping hand: Boys and Girls Clubs of America, Atlanta, GA; 1993. 75p. p. |
|  | 52. Davis RH. Intergenerational dialogues project: audience impact study: University of Southern California, Ethel Percy Andrus Gerontology Center, Media Projects Office, Los Angeles, CA; 1981. 102p. p. |
|  | 53. De Schutter B, Roberts AR. A workshop on intergenerational game concept design and paper prototyping. Gerontechnology. 2016;15. |
|  | 54. de Souza EM. Intergenerational relationship in health promotion programme: the experience of Brazil. Journal of Intergenerational Relationships. 2004;2(2):95-100. |
|  | 55. Deutchman DE, Bruno KA, Jarrott SE. Young at heart: intergenerational activities involving persons with dementia. Activities Directors’ Quarterly for Alzheimer’s and Other Dementia Patients. 2003;4(2):27-36. |
|  | 56. DeVore S, Aeschlimann E. Song partners for kindergartens: An intergenerational program in Switzerland. Journal of Intergenerational Relationships. 2016;14(1):60-4. |
|  | 57. Disch R. Young, the old, and the life review: report on a Brookdale project. Journal of Gerontological Social Work. 1988;12(3-4):125-35. |
|  | 58. Dlugokinski E, Rest S. Facilitating mutually supportive linkages between generations: GIFT after two years. Lifelong Learning. 1982;5(6):12. |
|  | 59. Dreibelbis TD, George DR. An intergenerational teaching kitchen: Reimagining a senior center as a shared site for medical students and elders enrolled in a culinary medicine course. Journal of Intergenerational Relationships. 2017;15(2):174-80. |
|  | 60. Drenning S, Getz L. Computer Ease. Phi Delta Kappan. 1992;73(6):471-2. |
|  | 61. Drury L, Abrams D, Swift HJ. Making intergenerational connections: an evidence review. 2017. |
|  | 62. du Toit S, Lok Yi C, Angelou K, McDonnell C, Lee-Fay L. Gold Soul companionship. Australian Journal of Dementia Care. 2019;8(4):15-7. |
|  | 63. DuBois DL, Portillo N, Rhodes JE, Silverthorn N, Valentine JC. How Effective Are Mentoring Programs for Youth? A Systematic Assessment of the Evidence. Psychological Science in the Public Interest. 2011;12(2):57-91. |
|  | 64. Duncan R, Whitley E, McKenzie P, Sledjeski S. From time to time: a record of young children’s relationships with the aged: P.K. Yonge Laboratory School, College of Education, University of Florida, Gainesville, FL; 1976. 98p. p. |
|  | 65. Dye J. Meet generation C: Creatively connecting through content. Econtent. 2007;30(4):38-43. |
|  | 66. Elaine S. Promoting intergenerational programmes: where is the evidence to inform policy and practice? Evidence and Policy. 2009;5(4):471-88. |
|  | 67. Eligator R, Schrading B, Browne M. New way for schools: intergenerational teaming of adults in classrooms for children: Shady Lane School, Pittsburgh, PA; 1980. 35p. p. |
|  | 68. Emma G. Learning from intergenerational housing projects in the USA. 2018:79. |
|  | 69. Fand F. Adopt-a-Grandparent Program Teaches about Life. Middle School Journal. 1996;27(5):22-8. |
|  | 70. Feldman S, Mahoney H, Seedsman T. Education for positive ageing: a partnership model for effecting sustainable outcomes. Education and Ageing. 2002;17(1):7-23. |
|  | 71. Fitzpatrick C, Musser A, Mosqueda L, Boker J, Prislin M. Student Senior Partnership Program: University of California Irvine School of Medicine. Gerontology & geriatrics education. 2006;27(2):25-35. |
|  | 72. Foret CM, Carter MJ, Nezey I, Ozene G. Project PIER: anchor and bridges into the community. Ageing International. 1997;24(2-3):112-9. |
|  | 73. Foster KB. Creating a Child Care Center in a Nursing Home and Implementing an Intergenerational Program. 1997. |
|  | 74. Foster KB. Intergenerational Programming: The Impact on Children and Elders. 1997. |
|  | 75. Frankland R, Conder S. Preventing lost generations: using intergenerational work to help young people. Quality in Ageing & Older Adults. 2012;13(4):282-5. |
|  | 76. Freedman M. Fostering Intergenerational Relationships for at-Risk Youth. Children Today. 1989;18(2):10-5. |
|  | 77. Freeman NK, King S. Service learning in preschool: An intergenerational project involving five-year-olds, fifth graders, and senior citizens. Early Childhood Education Journal. 2001;28(4):211-7. |
|  | 78. Friedman BM, Godfrey F. Intergenerational exercise addresses the public health issue of obesity. Journal of Intergenerational Relationships. 2007;5(1):79-94. |
|  | 79. Fujiwara Y. LONG-TERM EFFECTS OF AN INTERGENERATIONAL PROGRAM ON HEALTH AND WELL-BEING OF OLDER ADULTS. Gerontologist. 2016;56:9-. |
|  | 80. Fujiwara Y, Ohba H, Yasunaga M, Nonaka K, Nishi M, Murayama Y, et al. INTERGENERATIONAL HEALTH PROMOTION PROGRAM BY OLDER ADULTS -THREE-YEAR EXPERIENCE AND EFFECTS. Gerontologist. 2012;52:176-. |
|  | 81. Fujiwara Y, Sakurai R, Yasunaga M, Murayama Y, Suzuki H, Nishi M, et al. Long-term effects of an intergenerational program on functional capacity in older adults: evidence from a seven-year follow-up of the REPRINTS study. Gerontologist. 2015;55. |
|  | 82. Fujiwara Y, Uchida H, Shinkai S. A SCHOOL VOLUNTEERING PROGRAM BY OLDER ADULTS FOR URBAN CHILDREN IN JAPAN. Gerontologist. 2013;53:448-. |
|  | 83. Gabriele H, Christoph N. An intergenerational cooking activity: Benefits for residents and children. European Geriatric Medicine. 2019;10(Supplement 1):S153. |
|  | 84. George DR, Whitehouse PJ. Intergenerational volunteering and quality of life for persons with mild-to-moderate dementia: results from a 5-month intervention study in the United States. Journal of the American Geriatrics Society. 2010;58(4):796-7. |
|  | 85. Gita P. Supported independent living: communal and intergenerational living in the Netherlands and Denmark. 2019:56. |
|  | 86. Good GS. Intergenerational programming in Canada: a situational review: Canadian Institute of Child Health, Ottawa, Ontario, Canada; 1995. 69p. p. |
|  | 87. Goyer A. Intergenerational shared-site programs. Generations. 1998;22(4):79-80. |
|  | 88. Granville G, Laidlaw J. A partnership of trust: young offenders supporting older people in care settings. 2000. |
|  | 89. Greenblatt F. Adopt-A-Grandchild program: Improving attitudes of adolescents toward the aged. Activities, Adaptation & Aging. 1982;3(1):21-5. |
|  | 90. Grindell C, Gerrish K, Bissell P, Mawson S. iStep: Evaluation of a novel social innovation to encourage intergenerational physical activity...The 35th Scientific Meeting of the Physiotherapy Research Society 16 April 2016, University of Leicester, UK. International Journal of Therapy & Rehabilitation. 2016;23(6):S275-S. |
|  | 91. Grogan M. The Friends of Seniors Foundation: ‘fostering friendships between teens and seniors through visits and activities’. Journal of Intergenerational Relationships. 2004;2(1):97-9. |
|  | 92. Guastadisegni PE, Simon JL, Neuwelt J, Camicioli R, Mueller R, Neuwelt EA. Innovative intergenerational prevention programming for older adults and adolescents. Journal of the American Geriatrics Society. 2000;48(1):105-6. |
|  | 93. Gubner J, Allison TA. Transforming undergraduate student perceptions of dementia through collaborative filmmaking: Intergenerational films about music and memory. Journal of the American Geriatrics Society. 2019;67(Supplement 1):S5. |
|  | 94. Guerrero LR, Jimenez P, Tan Z. TimeOut@UCLA: An intergenerational respite care and workforce development program. Journal of Intergenerational Relationships. 2017;15(3):290-4. |
|  | 95. Gundling R. Bridging the Gap Between Generations: An Intergenerational Program Between a Public School, Senior Center and Two Private Nursing Facilities 1986. |
|  | 96. Hannon PO. An investigation of the impact of short-term quality intergenerational contact on children’s attitudes toward older adults: Pennsylvania State University; 2004. |
|  | 97. Hayden CS. Elder Campers Helping Others: The ECHO project: A unique intergenerational model. Activities, Adaptation & Aging. 1988;11(1):11-9. |
|  | 98. Hayden CS. Elder Campers Helping Others--the ECHO Project: a unique intergenerational model. Activities, Adaptation and Aging. 1988;11(1):11-9. |
|  | 99. Hayman LL. Dance for Health: An Intergenerational Program to Increase Access to Physical Activity. MCN: The American Journal of Maternal Child Nursing. 2018;43(1):56-. |
|  | 100. Hegeman C, Buzanowski DA, Davenport A. LESSONS LEARNED FROM A THREE YEAR INTERGENERATIONAL SERVICE LEARNING PROGRAM. Gerontologist. 2009;49:339-. |
|  | 101. Henkin NZ. Time out, an intergenerational approach to respite care: executive summary: Temple University, Center for Intergenerational Learning, Institute on Aging, Philadelphia, PA; 1989. 10p. p. |
|  | 102. Hernandez A. Intergenerational mentoring program for Hispanic women: A grant writing project [M.S.W.]. Ann Arbor: California State University, Long Beach; 2007. |
|  | 103. Heydon RM. The De-Pathologization of Childhood, Disability and Aging in an Intergenerational Art Class: Implications for Educators. Journal of Early Childhood Research. 2005;3(3):243-68. |
|  | 104. Hill D. Promoting intergenerational programs: triads with youth, elderly, and students, a case example. Journal of Gerontological Social Work. 1987;10(3-4):155-65. |
|  | 105. Hill H. Intergenerational dance/movement program in Melbourne, Australia. Journal of Intergenerational Relationships. 2007;5(1):97-101. |
|  | 106. Hirn D. Children’s Family Center: a shared-site intergenerational childcare program. Journal of Intergenerational Relationships. 2007;5(2):119-25. |
|  | 107. Hirsch AL, Shen MJ, Lachs MS. Intergenerational sharing of tea & poetry enhances mood in the elderly. Journal of the American Geriatrics Society. 2018;66(Supplement 2):S306. |
|  | 108. Hirsch GA, Hirsch A, Shen M, Czaja S, Lachs M. A Program of Intergenerational Sharing of Tea and Poetry with the Elderly Successfully Translated onto a Virtual Platform during the COVID-19 Pandemic. Journal of the American Geriatrics Society. 2021;69:S239-S. |
|  | 109. Hirsch GA, Shen MJ, Lachs M. Replication of a Community Program of Intergenerational Sharing of Tea and Poetry with the Elderly. Journal of the American Geriatrics Society. 2020;68:S281-S2. |
|  | 110. Hoang P, Whaley C, Thompson K, Ho V, Rehman U, Boluk K, et al. Evaluation of an Intergenerational and Technological Intervention for Loneliness: Protocol for a Feasibility Randomized Controlled Trial. Journal of Medical Internet Research. 2021;23(2):N.PAG-N.PAG. |
|  | 111. Horan K, Perkinson MA. Reflections on the dynamics of a student-organized intergenerational visiting program to promote social connectedness. Journal of Intergenerational Relationships. 2019;17(3):396-403. |
|  | 112. Husser E, Jarrott S. Virginia Tech Intergenerational Programs: Growing together. Gerontologist. 2005;45:579-80. |
|  | 113. Irct20190918044816N. The effect of intergenerational interaction on the elderlyâ€™s health. http://wwwwhoint/trialsearch/Trial2aspx?TrialID=IRCT20190918044816N1. 2020. |
|  | 114. Jarrott SE, Gigliotti CM, Smock SA. Where do we stand? Testing the foundation of a shared site intergenerational program. Journal of Intergenerational Relationships. 2006;4(2):73-92. |
|  | 115. Jarrott SE, Weaver RH. PILOTING A COMMUNITY-BASED PARTICIPATORY APPROACH TO SUSTAINING INTERGENERATIONAL PROGRAMS. Gerontologist. 2013;53:56-. |
|  | 116. Johnson EN, Alghanim F, Nothelle S, Ayyala M, Rios R. Voices of intergenerational community experiences (project voice): A co-learning educational initiative with resident physicians and community members. Journal of General Internal Medicine. 2018;33(2 Supplement 1):753-4. |
|  | 117. Jones M. Walking the talk, together. Northwest Public Health. 2007;24(2):18-9. |
|  | 118. Kaplan M. Benefits of intergenerational community service projects: implications for promoting intergenerational unity, community activism, and cultural continuity. Journal of Gerontological Social Work. 1997;28(3):211-28. |
|  | 119. Kaplan M, Greenwood-Junkermeier H, Bradley L. Unlocking the Potential of Older Adult Volunteers: The Intergenerational Leadership Institute Model as a Resource for Bolstering Extension. Journal of Extension. 2019;57(5). |
|  | 120. Kaplan M, Kusano A, Tsuji I, Hisamichi S. Intergenerational programs: support for children, youth, and elders in Japan: State University of New York Press, Albany, NY; 1998. 278p. p. |
|  | 121. Keller MJ. Intergenerational sharing: Teens and Elderly for the Arts (TEA). Journal of Applied Gerontology. 1990;9(3):312-24. |
|  | 122. Kennedy O, Boland P. 100 The Factors Which Impact Non-Familial Intergenerational Interaction within Public Space: An Integrative Review. Age and Ageing. 2019;48. |
|  | 123. King SP, Lauder R. Active living and learning: A multifaceted intergenerational program. Journal of Intergenerational Relationships. 2016;14(2):151-5. |
|  | 124. Kleijberg M. Navigating power dynamics in engaging communities in end-of-life issues: Analysis of the process of developing a community-based intergenerational arts initiative about death and loss as part of the Swedish DoBra Research Program. Progress in Palliative Care. 2020;28(2):162. |
|  | 125. Kleijberg M, Ahlberg BM, Hilton R, Tishelman C. Reflections from children and older adults participating in community-based arts initiatives about dying, death, and loss in sweden. Palliative Care and Social Practice. 2021;15:20. |
|  | 126. Krause EB, Bauman PM. Intergenerational programming for foster-adoptive families: creating community at Hope Meadows. Journal of Intergenerational Relationships. 2003;1(1):17-28. |
|  | 127. Krout JA, Pogorzala CH. An intergenerational partnership between a college and congregate housing facility: how it works, what it means. The Gerontologist. 2002;42(6):853-8. |
|  | 128. Kuehne VS. Building intergenerational communities through research and evaluation. Generations. 1998;22(4):82-7. |
|  | 129. Kuehne VS. State of our art: intergenerational program research and evaluation: part one. Journal of Intergenerational Relationships. 2003;1(1):145-61. |
|  | 130. Lance C, James J, Watson M. Can bringing children and older people together, in an acute hospital setting, bring beneficial effects to all those involved. Archives of Disease in Childhood. 2019;104(Supplement 2):A21-A2. |
|  | 131. Lapolla D, Kothari K, McIntyre C, Javier NM. The Creation of an Intergenerational Legacy Project through a Virtual Platform as a Tool to Deconstruct Ageist Attitudes among Pre-Clinical Medical Students. Journal of the American Geriatrics Society. 2021;69:S273-S. |
|  | 132. Leduc RD, Turcotte S, Dionne B, Grenier S, Ladjadj F, Filiatrault J. LES ACTIVITÉS INTERGÉNÉRATIONNELLES EN TANT QUE STRATÉGIE DE PROMOTION DE LA SANTÉ DES AÎNÉS: UNE ÉTUDE DE LA PORTÉE. Revue Francophone de Recherche en Ergotherapie. 2020;6(2):33-73. |
|  | 133. Liu L, Lin Y. INTERGENERATIONAL SERVICE PROGRAM: CONSTRUCTION OF COMMUNITY SOCIAL SUPPORT NETWORK FOR THE ELDERLY. Gerontologist. 2012;52:556-. |
|  | 134. Liu S-T, Kaplan MS. An Intergenerational Approach for Enriching Children’s Environmental Attitudes and Knowledge. Applied Environmental Education and Communication. 2006;5(1):9-20. |
|  | 135. Loe M. The digital life history project: intergenerational collaborative research. Gerontology & geriatrics education. 2013;34(1):26-42. |
|  | 136. Lynott P, Merola P, Ruckert E. Changes in 4th graders’ drawings after participating in an intergenerational program. Gerontologist. 2004;44:231-. |
|  | 137. MacDonald MJ. Investigation of intergenerational relationships occurring within a shared reading program: UMI Dissertation Services, ProQuest Information and Learning, Ann Arbor, MI; 2003. |
|  | 138. MacDonald MJ. An investigation of intergenerational relationships occurring within a shared reading program [Ph.D.]. Ann Arbor: University of Victoria (Canada); 2003. |
|  | 139. Mano M. Role of Intergenerational Mentoring for Supporting Youth Development: An Examination of the “Across Ages” Program in the U. S. Educational Studies in Japan: International Yearbook. 2007(2):83-94. |
|  | 140. Martinson M, Minkler M, Garcia A. Honoring, Training, and Building a Statewide Network of Elder Activists: The California Senior Leaders Program (2002–2012). Journal of Community Practice. 2013;21(4):327-55. |
|  | 141. Matsuzawa H, Miyamoto N, Tsuboyama Y, Kurokawa Y. MULTIPLE PROGRAMS OF INTERGENERATIONAL REMINISCENCE FOR OLDER ADULTS AND THE YOUNG GENERATION. Gerontologist. 2010;50:87-. |
|  | 142. Mehrotra CM, Bail ZL, Haglin LM. BRINGING GENERATIONS TOGETHER TO STRENGTHEN RURAL COMMUNITIES AND INDIAN RESERVATIONS. Gerontologist. 2012;52:120-. |
|  | 143. Mehrotra CM, Bail ZL, Haglin LM. SUMMER INTERNSHIPS FOR UNDERGRADUATE STUDENTS IN RURAL INTERGENERATIONAL PROGRAMS. Gerontologist. 2013;53:330-. |
|  | 144. Melvin CS, Ryder KH. Among Friends: an intergenerational program for Alzheimer’s patients. VNA of Burlington, Vermont. Caring: National Association for Home Care magazine. 1989;8(8):26-8. |
|  | 145. Mena C. EXPLORING THE BENEFITS OF Intergenerational Programs. Exceptional Parent. 2017;47(3):20-1. |
|  | 146. Meshel DS. The contact hypothesis and the effects of intergenerational contact on adolescents’ attitudes and stereotypes toward older people [Ph.D.]. Ann Arbor: Texas Tech University; 1997. |
|  | 147. Mestre A, Llorente K, Lafarga O, Tuyn D, Montalli S, Montalli L. ESeniors: An intergenerational model to enhance computer skills for older adults. American Journal of Geriatric Psychiatry. 2011;19(3 SUPPL. 1):S82. |
|  | 148. Miller WR, et al. The elderly and the young: a cooperative endeavor. Journal of Gerontological Social Work. 1991;17(1/2):93-104. |
|  | 149. Molina-Luque F, Casado N, Stončikaitė I. University stakeholders, intergenerational relationships and lifelong learning: a European case study. Educational Gerontology. 2018;44(12):744-52. |
|  | 150. Mosor E, Waldherr K, Hubel U, Pinter-Theiss V, Stamm T. Moving Generations - Evaluation of an intergenerational health promotion program based on psychomotricity. Zeitschrift Fur Gerontologie Und Geriatrie. 2017;50:S20-S. |
|  | 151. Muir KB. Measuring the benefits of mentoring for foster grandparents: A research note. Special Issue: Elderly volunteerism. 2006;32(5):379-87. |
|  | 152. Murayama Y, Yasunaga M, Takeuchi R, Nonaka K, Ohba H, Kuraoka M, et al. THE EFFECT OF INTERGENERATIONAL PROGRAMS FOR PRIMARY SCHOOL STUDENTS THROUGH AN INTERGENERATIONAL PROGRAM WITH READING PICTURE BOOKS. Gerontologist. 2012;52:72-. |
|  | 153. Ng J. Promoting intergenerational relationships through table tennis. Journal of Intergenerational Relationships. 2005;3(1):89-92. |
|  | 154. Nichols AH. LINC Project: an intergenerational statewide collaborative project. Journal of Intergenerational Relationships. 2003;1(2):33-46. |
|  | 155. Nickse RS. The Noises of Literacy: An Overview of Intergenerational and Family Literacy Programs. 1989. |
|  | 156. Nishita C. THE ONE MILE PROJECT-AGING AND INTERGENERATIONAL EDUCATION IN HIGH SCHOOL. Gerontologist. 2015;55:301-. |
|  | 157. Olson MN. INTERGENERATIONAL ACTIVITIES PROGRAM (IGAP): PROMOTING MENTAL HEALTH IN ADOLESCENTS AND OLDER ADULTS IN THERMOPOLIS, WYOMING. Journal of Investigative Medicine. 2018;66(1):139-. |
|  | 158. Oosman S, Abonyi S. Full circle-do intergenerational approaches hold promise for preventing and managing obesity among aboriginal populations? Canadian Journal of Diabetes. 2013;37(SUPPL. 2):S287. |
|  | 159. Owen M, Wright P. Intergenerational Entrepreneurship in an Educational Setting. 1994. |
|  | 160. Parker JM. Building Bridges in Midtown Manhattan: An Intergenerational Literacy Program. Urban Education. 1989;24(1):109-15. |
|  | 161. Parkinson D, Turner J. Alleviating social isolation through intergenerational programming: DOROT’s Summer Teen Internship Program. Journal of Intergenerational Relationships. 2019;17(3):388-95. |
|  | 162. Parson F. Finding common ground: intergenerational programs connect LGBT elders, youth. Aging Today. 2014;35(3):1-3. |
|  | 163. Peterat L, Mayer-Smith J. Farm friends: Exploring intergenerational environmental learning. Special Issue: Global challenges-future directions: Intergenerational programs, research, and social policy. 2006;4(1):107-16. |
|  | 164. Pinazo S, Montoro-Rodriguez J. An intergenerational program, ‘Nau Gran,’ at the University of Valencia (Spain). Journal of Intergenerational Relationships. 2003;1(2):103-4. |
|  | 165. Pogorzala CH, Krout JA. A comprehensive Intergenerational programmatic partnership: Initial issues and outcomes. Gerontology & Geriatrics Education. 2001;21(4):39-54. |
|  | 166. Potts DC, Carden KD, Myrick J, Gately S, Allen RS, Broman E, et al. Bringing art to life: An intergenerational service learning course and visual art therapy intervention for college students, persons with Alzheimer’s disease and dementia, and caregivers. Alzheimer’s and Dementia. 2015;11(7 SUPPL. 1):P581. |
|  | 167. Powers WG, Bailey-Hughes B, Ranft M. Senior citizens as educational resources. Educational Gerontology. 1989;15(5):481-7. |
|  | 168. Predny ML, Relf D. Horticulture Therapy Activities for Preschool Children, Elderly Adults, and Intergenerational Groups. Activities, Adaptation & Aging. 2004;28(3):1-18. |
|  | 169. Ramón-Gancedo F. Gent Gran, Gent Petita, A Shared Experience. Journal of Intergenerational Relationships. 2018;16(1/2):190-5. |
|  | 170. Roodin P, Brown LH, Shedlock D. Intergenerational Service-Learning: A Review of Recent Literature and Directions for the Future. Gerontology & Geriatrics Education. 2013;34(1):3-25. |
|  | 171. Rosebrook V. Intergenerational Connections Enhance the Personal/Social Development of Young Children. International Journal of Early Childhood. 2002;34(2):30-41. |
|  | 172. Rossberg-Gempton IE, Poole GD. An intergenerational creative dance program for children of frail older adults. Gerontology & Geriatrics Education. 1999;20(2):49-68. |
|  | 173. Roth DE. Young adult college students reflect on their interaction with frail elders in culture change: in light of world religions. Journal of Intergenerational Relationships. 2004;2(1):29-45. |
|  | 174. Rothstein DG. DEVELOPING A VOLUNTARY, NEIGHBORHOOD INTERGENERATIONAL PROGRAM. Journal of Gerontological Social Work. 1983;6(1):99-106. |
|  | 175. Sauer P, Hahn SJ, Beanblossom K, Kinney JM, Lokon E. WELL- AND ILL-BEING: RESULTS FROM VIDEO DATA ANALYSES OF AN INTERGENERATIONAL ART PROGRAM FOR PEOPLE WITH DEMENTIA. Gerontologist. 2012;52:653-. |
|  | 176. Scirpo AC. AN INTERGENERATIONAL PROGRAM AND REMINISCING: INTERVENTIONS WITH A NURSING HOME RESIDENT [M.S.W.]. Ann Arbor: Southern Connecticut State University; 1987. |
|  | 177. Seeley C, Porter S. Promoting intergenerational understanding through Community Philosophy. Findings. 2008. |
|  | 178. Shippee TP, Schafer M, Pallone K. Building the “bridge”: linking gerontology education to a local retirement community. Gerontology & geriatrics education. 2008;28(4):57-70. |
|  | 179. Sikora S. University of Arizona College of Medicine Optimal Aging Program: stepping in the shadows of successful aging. Gerontology and Geriatrics Education. 2006;27(2):59-67. |
|  | 180. Sowle AJ, Francis SL, Margrett JA, Franke WD. Utility of the Living (Well Through) Intergenerational Fitness and Exercise Program as a County-Delivered Extension Program. Journal of Extension. 2016;54(4). |
|  | 181. Springate I, Atkinson M, Martin K, National Foundation for Educational R. Intergenerational Practice: A Review of the Literature. LGA Research Report F/SR262. National Foundation for Educational Research; 2008. Report No.: 978-1-905314-86-7. |
|  | 182. Stead AL, Reuler E. A Preclinical Experience for Clinical Integration in a Transgenerational Community Partnership. Contemporary Issues in Communication Science & Disorders. 2016;43:299-305. |
|  | 183. Strande PO. School Grandparents in Norway. Journal of Intergenerational Relationships. 2006;4(3):113-4. |
|  | 184. Teasdale R. Best Practice: Testing the theory. Activities, Adaptation & Aging. 2012;36(1):79-82. |
|  | 185. Valerie K. The state of our art: intergenerational program research and evaluation: part two. Journal of Intergenerational Relationships. 2003;1(2):79-94. |
|  | 186. Yanez MA. “Mejor juntos” (better together!): intergenerational relationships in Baza: a cultural and environmental experience. Journal of Intergenerational Relationships. 2005;3(4):101-6. |
| Wrong Population | 1. Atkins R, Deatrick JA, Bowman C, Bolick A, McCurry I, Lipman TH. University-Community Partnerships Using a Participatory Action Research Model to Evaluate the Impact of Dance for Health. Behavioral sciences (Basel, Switzerland). 2018;8(12). |
|  | 2. Atkins R, Deatrick JA, Gage GS, Earley S, Earley D, Lipman TH. Partnerships to Evaluate the Social Impact of Dance for Health: A Qualitative Inquiry. Journal of community health nursing. 2019;36(3):124-38. |
|  | 3. Brown B, Dybdal L, Noonan C, Pedersen MG, Parker M, Corcoran M. Group Gardening in a Native American Community: A Collaborative Approach. Health Promotion Practice. 2020;21(4):611-23. |
|  | 4. Butler FR, Baghi H. Using the Internet to facilitate positive attitudes of college students toward aging and working with older adults. Journal of Intergenerational Relationships. 2008;6(2):175-89. |
|  | 5. Castro JL, González DA, Aguayo IH, Fernández EA. Perceptions Concerning Intergenerational Education from the Perspective of Participants. Educational Gerontology. 2014;40(2):138-51. |
|  | 6. Celdrán M, Serrat R, Villar F, Pinazo S, Solé C. The Experiences of Retired Managers Acting as Volunteers in an Entrepreneurial Mentoring Organization. Journal of Population Ageing. 2018;11(1):67-81. |
|  | 7. Chan CC, Ho W-c. Intensive community mentoring scheme in Hong Kong: Nurturing police-youth intergenerational relationships. Journal of Intergenerational Relationships. 2006;4(2):101-6. |
|  | 8. Conway C, Hodgman TM. College and Community Choir Member Experiences in a Collaborative Intergenerational Performance Project. Journal of Research in Music Education. 2008;56(3):220-37. |
|  | 9. Cordier R, Wilson NJ, Stancliffe RJ, MacCallum J, Vaz S, Buchanan A, et al. Formal intergenerational mentoring at Australian Men’s Sheds: a targeted survey about mentees, mentors, programmes and quality. Health & social care in the community. 2016;24(6):e131-e43. |
|  | 10. Cumming-Potvin WM, MacCallum J. Intergenerational Practice: Mentoring and Social Capital for Twenty-First Century Communities of Practice. McGill Journal of Education. 2010;45(2):305-23. |
|  | 11. Dauenhauer JA, Heffernan KM, Cesnales NI. Promoting intergenerational learning in higher education: Older adult perspectives on course auditing. Educational Gerontology. 2018;44(11):732-40. |
|  | 12. DuBois DL, Neville HA, Parra GR, Pugh-Lilly AO. Testing a new model of mentoring. New directions for youth development. 2002(93):21‐57. |
|  | 13. Duvall J, Zint M. A Review of Research on the Effectiveness of Environmental Education in Promoting Intergenerational Learning. Journal of Environmental Education. 2007;38(4):14-24. |
|  | 14. Ehlman K, Ligon M, Moriello G. The impact of intergenerational oral history on perceived generativity in older adults. Journal of Intergenerational Relationships. 2014;12(1):40-53. |
|  | 15. Elza D. Intergenerational interaction through reminiscence processes: a theoretical framework to explain attitude changes. Journal of Intergenerational Relationships. 2007;5(1):39-56. |
|  | 16. Ermer AE, York K, Mauro K. Addressing ageism using intergenerational performing arts interventions. Gerontology & geriatrics education. 2021;42(3):308-15. |
|  | 17. Ermis SA. An examination of the effects of an intergenerational reading project on home literacy experiences and the literacy development of preschool students [Ed.D.]. Ann Arbor: Texas A&M University - Kingsville; 1996. |
|  | 18. France MG. An examination of the effects of an intergenerational reading workshop on the listening comprehension of at-risk pre-kindergarten students. Dissertation Abstracts International Section A: Humanities and Social Sciences. 1992;52(12-A):4215-6. |
|  | 19. Grindell C, Mawson S, Gerrish K, Parker S, Bissell P. Exploring the acceptability and usability of a novel social innovation to encourage physical activity: The iStep prototype. Health & social care in the community. 2019;27(2):383-91. |
|  | 20. Hammrich PL, Richardson GM, Livingston B. Sisters in Science: An Intergenerational Science Program for Elementary School Girls. 1999. |
|  | 21. Hamon RR, Koch DK. Elder mentor relationship: an experiential learning tool. Educational Gerontology. 1993;19(2):147-59. |
|  | 22. Jarrott SE, Gigliotti CM, Smock SA. Programming. Where do we stand? Testing the foundation of a shared site intergenerational program. Journal of Intergenerational Relationships. 2006;4(2):73-92. |
|  | 23. Jocey Q, Claudia B. The power of songs: an evaluation of Plymouth music zone’s ‘Keep Singing, Keepsake’ project. 2014:48. |
|  | 24. John H, Olivia P, Amanda N. North London Cares and South London Cares evaluation: final report. 2019:27. |
|  | 25. Jourdan DE. Planning to reduce worry: Designing an intergenerational planning process to lessen relocation -related anxieties experienced by those displaced in the pursuit of a HOPE VI Revitalization Grant [Ph.D.]. Ann Arbor: The Florida State University; 2004. |
|  | 26. Julian M-R, Sacramento P. Evaluating social integration and psychological outcomes for older adults enrolled at a University intergenerational program. Journal of Intergenerational Relationships. 2005;3(3):65-81. |
|  | 27. Kelley LS. Minor children and adult care exchanges with community-dwelling frail elders in a St. Lucian village. The journals of gerontology Series B, Psychological sciences and social sciences. 2005;60(2):S62-73. |
|  | 28. Kelly JD. A study of intergenerational education in secondary vocational programs in Pennsylvania [Ph.D.]. Ann Arbor: The Pennsylvania State University; 1991. |
|  | 29. Kocarnik RA, Ponzetti JJ. The influence of intergenerational contact on child care participants’ attitudes toward the elderly. Child Care Quarterly. 1986;15(4):244-50. |
|  | 30. Krishnan S, Subbiah K, Khanum S, Chandra PS, Padian NS. An Intergenerational Women’s Empowerment Intervention to Mitigate Domestic Violence: Results of a Pilot Study in Bengaluru, India. Violence Against Women. 2012;18(3):346-70. |
|  | 31. Lally JR, Grossman CH. Exploring Employment Opportunities for the Elderly in Child Care Services: A Feasibility Study. Report No. 3. 1981. Report No.: 5. |
|  | 32. Lokon E, Li Y, Kunkel S. Allophilia: Increasing college students’ “liking” of older adults with dementia through arts-based intergenerational experiences. Gerontology & geriatrics education. 2020;41(4):494-507. |
|  | 33. Lokon E, Li Y, Parajuli J. Using art in an intergenerational program to improve students’ attitudes toward people with dementia. Gerontology & geriatrics education. 2017;38(4):407-24. |
|  | 34. Mason MV. A Qualitative Study Exploring the Implementation and Effects of Intergenerational Youth Ministry [Ph.D.]. Ann Arbor: Biola University; 2020. |
|  | 35. McClusky HY, Brahce C. COMPARATIVE SUPPORT ROLES FOR OLDER VOLUNTEERS AND NON-VOLUNTEERS IN SCHOOL LEARNING ACTIVITIES. Gerontologist. 1982;22:217-8. |
|  | 36. Osborne SS, Bullock JR. Intergenerational programming in action: befrienders. Educational Gerontology. 2000;26(2). |
|  | 37. Passey D. Intergenerational Learning Practices--Digital Leaders in Schools. Education and Information Technologies. 2014;19(3):473-94. |
|  | 38. Powell JV, et al. Effects of Intergenerational Tutoring and Related Variables on Reading and Mathematics Achievement of Low Socioeconomic Children. Journal of Experimental Education. 1987;55(4):206-11. |
|  | 39. Putnam R. Beyond the classroom: An exploration of student engagement in a campus child care center. Dissertation Abstracts International: Section B: The Sciences and Engineering. 2020;81(3-B):No-Specified. |
|  | 40. Ralston PA, Furlow J, Brickler-Hart C, Baker L, Austin D, Ford CA, et al. The Community Wellness Program: an intergenerational seminar for African Americans. Journal of health care for the poor and underserved. 2007;18(1):21-7. |
|  | 41. Ransdell LB, Robertson L, Ornes L, Moyer-Mileur L. Generations Exercising Together to Improve Fitness (GET FIT): a pilot study designed to increase physical activity and improve health-related fitness in three generations of women. Women & health. 2004;40(3):77-94. |
|  | 42. Rubin JD, Scanlon M, Cechony A, Chen K. “Here I can just be myself”: How youth and adults collaboratively develop an identity-safe community across difference. Journal of community psychology. 2021;49(5):1024-43. |
|  | 43. Scott JP, Reifman A, Mulsow M, Feng D. Program evaluation of “Young at Heart”: examining elderly volunteers’ generativity. Journal of Intergenerational Relationships. 2003;1(3):25-33. |
|  | 44. Segrist K. Assessing impact of service-learning project on older, isolated adults in rural America. Journal of Intergenerational Relationships. 2004;2(2):51-66. |
|  | 45. Teffel M. Bringing the young and old together: how to connect international youth work with intergenerational practice. Working with Older People: Community Care Policy & Practice. 2011;15(2):53-7. |
|  | 46. Wallace-Fierte LN. Intergenerational programs as a pathway to positive intergenerational relationships and community involvement. Dissertation Abstracts International Section A: Humanities and Social Sciences. 2016;77(1-A(E)):No-Specified. |
|  | 47. Wilsey SA, Arnold NY, Criado MM, Mykita A. Experiential teaching in an adult development course: promoting an understanding of intergenerational interactions. Journal of prevention & intervention in the community. 2013;41(2):82-8. |
| Wrong Intervention | 1. Arcos-Alonso A, Ortega ÁE, Arcos Alonso A. Intergenerational Service-Learning, Sustainability and University Social Responsibility: A Pilot Study. Cypriot Journal of Educational Sciences. 2020;15(6):1629-41. |
|  | 2. B PM, et al. Aging well in an intentional intergenerational community: meaningful relationships and purposeful engagement. Journal of Intergenerational Relationships. 2007;5(2):7-25. |
|  | 3. Brady B, Dolan P, Canavan J. ‘He told me to calm down and all that’: a qualitative study of forms of social support in youth mentoring relationships. Child & Family Social Work. 2017;22(1):266-74. |
|  | 4. Chonody J, Wang D. Connecting older adults to the community through multimedia: An intergenerational reminiscence program. Activities, Adaptation & Aging. 2013;37(1):79-93. |
|  | 5. Corrigan T, McNamara G, O’Hara J. Intergenerational Learning: A Valuable Learning Experience for Higher Education Students. Eurasian Journal of Educational Research. 2013(52):117-36. |
|  | 6. Flora PK. Intergenerational programming: examining the role of physical activity: UMI Dissertation Services, ProQuest Information and Learning, Ann Arbor, MI; 2006. |
|  | 7. Garrett SE. A relative efficacy study of advanced training effects on school-based youth mentors’ attitudes and experience in the program. Dissertation Abstracts International: Section B: The Sciences and Engineering. 2014;75(4-B(E)):No-Specified. |
|  | 8. Granacher U, Muehlbauer T, Gollhofer A, Kressig RW, Zahner L. An intergenerational approach in the promotion of balance and strength for fall prevention - a mini-review. Gerontology. 2011;57(4):304-15. |
|  | 9. Gusul M. Intergenerational theatre and the role of play [M.A.]. Ann Arbor: University of Alberta (Canada); 2009. |
|  | 10. Heffernan K, Cesnales N, Dauenhauer J. Creating intergenerational learning opportunities in multigenerational college classrooms: Faculty perceptions and experiences. Gerontology & geriatrics education. 2019:1-14. |
|  | 11. Heydon RM. Making Meaning Together: Multi-Modal Literacy Learning Opportunities in an Inter-Generational Art Programme. Journal of Curriculum Studies. 2007;39(1):35-62. |
|  | 12. Kaplan M, Liu S-T, Hannon P. Intergenerational Engagement in Retirement Communities: A Case Study of a Community Capacity-Building Model. Journal of Applied Gerontology. 2006;25(5):406-26. |
|  | 13. Kaplan MS. An intergenerational approach to community education and action: A case study [Ph.D.]. Ann Arbor: City University of New York; 1991. |
|  | 14. Kaplan MS, Weikert B, Scholl J, Rushton M. Intergenerational Panels at Centennial Events: Stimulating Discussion about Continuity and Change in the 4-H Program. Journal of Extension. 2013;51(1). |
|  | 15. Kogan LR, Schoenfeld-Tacher RM. Participation in an Intergenerational Service Learning Course and Implicit Biases. Educational Gerontology. 2018;44(2-3):90-8. |
|  | 16. Krout JA, McKernan P. The impact of gerontology inclusion on 12th grade student perceptions of aging, older adults and working with elders. Gerontology & geriatrics education. 2007;27(4):23-40. |
|  | 17. Kuehne VS. A comparative study of children’s extrafamilial intergenerational relations [Ph.D.]. Ann Arbor: Northwestern University; 1990. |
|  | 18. Kusano E, Ono M, Hayakawa K. Influence of support by elderly persons on Japanese mothers’ child care-related stress. Nursing & Health Sciences. 2010;12(2):182-90. |
|  | 19. Lawton PH. Artstories: Perspectives on intergenerational learning through narrative construction among adolescents, middle aged, and older aged adults [Ed.D.]. Ann Arbor: Teachers College, Columbia University; 2004. |
|  | 20. Lee Y, Kim Y-M, Bronstein L, Fox V. Older adult volunteers in intergenerational programs in educational settings across the globe. Educational Gerontology. 2021;47(6):247-56. |
|  | 21. Leviton D, Maria LS. Adults health and developmental program: descriptive and evaluative data. Gerontologist. 1979;19(6):234-43. |
|  | 22. Li Q, Kaplan M, Thang LL. Intergenerational Programs and Practices in China: Results Based on a Web Search Method: Research. Journal of Intergenerational Relationships. 2020;18(1):1-16. |
|  | 23. Lin Y-C, Dai Y-T, Huang L-H, Wang S-C, Huang G-S. Creative approach for successful aging: A pilot study of an intergenerational health promotion program. Geriatrics & gerontology international. 2017;17(11):1799-807. |
|  | 24. LoBuono DL, Leedahl SN, Maiocco E. Teaching Technology to Older Adults: Modalities Used by Student Mentors and Reasons for Continued Program Participation. Journal of gerontological nursing. 2020;46(1):14-20. |
|  | 25. Lovell EDn, Casey M, Randall L, Isaacson C, Bell M, Fox H, et al. Intergenerational Exchange: Undergraduate Researchers’ Learning and Listening Enhanced through Older Adults’ Entertainment Preferences. Educational Gerontology. 2018;44(7):469-77. |
|  | 26. Lovell EDn, Jordan KE, Scott R, Booth S, Ericson J, Strutzel R, et al. Intergenerational Undergraduate Gerontological Research Suggested Increased Commitment and Reduced Apprehensions to Learning. Adult Higher Education Alliance; 2018. |
|  | 27. Lva Lancaster-Lebanon Literacy Council LPA. Intergenerational Literacy. A 1989-90 Pennsylvania Department of Education Adult Education Division 353 Project. 1990. |
|  | 28. Moody E, Phinney A. A Community-Engaged Art Program for Older People: Fostering Social Inclusion. Canadian Journal on Aging. 2012;31(1):55-64. |
|  | 29. Morris D, Shaw B, Perney J. HELPING LOW READERS IN GRADE-2 AND GRADE-3 - AN AFTER-SCHOOL VOLUNTEER TUTORING PROGRAM. Elementary School Journal. 1990;91(2):133-50. |
|  | 30. Nagel J, Cimbolic P, Newlin M. EFFICACY OF ELDERLY AND ADOLESCENT VOLUNTEER COUNSELORS IN A NURSING-HOME SETTING. Journal of Counseling Psychology. 1988;35(1):81-6. |
|  | 31. Netragaonkar R, Swami AA, Swami SA. A cross sectional study to evaluate tree plantation and gardening as an intergenerational bonding activity amongst geriatric population. Indian Journal of Public Health Research and Development. 2020;11(3):1007-12. |
|  | 32. Nishi-Strattner M, Myers JE. Attitudes toward the elderly: An intergenerational examination. Educational Gerontology. 1983;9(5-6):389-97. |
|  | 33. Oster RT, Toth EL, Bell RC, Committee EFNCA. Wicohkamakew (“S/he Helps Someone”): A Qualitative Description of Experiences with a Community-derived Elders Mentoring Program for Indigenous Parents-to-be. Progress in community health partnerships: research, education, and action. 2021;15(2):177-87. |
|  | 34. Osterkamp LB, Chapin RK. Community-based volunteer home-repair and home-maintenance programs for elders: an effective service paradigm? Journal of Gerontological Social Work. 1995;24(1-2):55-75. |
|  | 35. Perla R, Tim B, Tim V. Ageing Better in Camden: interim evaluation report. 2018:107. |
|  | 36. Pynoos J, et al. Intergenerational Neighborhood Networks: A Basis for Aiding the Frail Elderly. Gerontologist. 1984;24(3):233-7. |
|  | 37. Raposa EB, Rhodes J, Stams GJJM, Card N, Burton S, Schwartz S, et al. The Effects of Youth Mentoring Programs: A Meta-analysis of Outcome Studies. Journal of youth and adolescence. 2019;48(3):423-43. |
|  | 38. Roberts E, Richeson N, Thornhill JT, Corwin SJ, Eleazer GP. The Senior Mentor Program at the University of South Carolina School of Medicine: an innovative geriatric longitudinal curriculum. Gerontology & geriatrics education. 2006;27(2):11-23. |
|  | 39. Roberts SJM. Creating an intergenerational support system in an African American church. Dissertation Abstracts International Section A: Humanities and Social Sciences. 2006;67(6-A):2193. |
|  | 40. Rupcic N. Intergenerational Learning and Knowledge Transfer--Challenges and Opportunities. Learning Organization. 2018;25(2):135-42. |
|  | 41. Senteio CR. Investigating the Enduring Impact of a Community-Based Health Education Program to Promote African American Elders’ Use of Technology Designed to Support Chronic Disease Self-Management. Geriatrics (Basel, Switzerland). 2018;3(4). |
| Wrong Outcomes | 1. Becker Fiegeles J. Reciprocity in intergenerational exchange: impact on seniors: UMI Dissertation Services, ProQuest Information and Learning, Ann Arbor, MI; 2006. |
|  | 2. Firman JP, Gelfand DE, Ventura C. Students as resources to the aging network. Gerontologist. 1983;23(2):185-91. |
|  | 3. Heydon R, McKee L, Daly B. iPads and Paintbrushes: Integrating Digital Media into an Intergenerational Art Class. Language and Education. 2017;31(4):351-73. |
|  | 4. Hubbard TE. Benefits of engaging older volunteers: A study of intergenerational initiatives in the public schools of the mid -Hudson Valley [Ed.D.]. Ann Arbor: University of Kansas; 2001. |
|  | 5. Karasik RJ. Reflecting on reflection: capitalizing on the learning in intergenerational service-learning. Gerontology & geriatrics education. 2013;34(1):78-98. |
|  | 6. Kleijberg M, Hilton R, Ahlberg BM, Tishelman C. Play Elements as Mechanisms in Intergenerational Arts Activities to Support Community Engagement with End-of-Life Issues. Healthcare (Basel, Switzerland). 2021;9(6). |
|  | 7. Kleijberg M, Hilton R, Ahlberg BM, Tishelman C. Using elements of play in arts activities to engage communities with end-of-life issues. Palliative Care and Social Practice. 2021;15:21. |
|  | 8. Kovacs PJ, Lee J. Developing a community-university partnership for intergenerational programming: Relationship building is key. Journal of Intergenerational Relationships. 2010;8(4):406-11. |
|  | 9. La Porte AM. Intergenerational Art Education: Building Community in Harlem. Journal of Social Theory in Art Education. 2002;22:51-71. |
|  | 10. Lindsay A. Enhancing Intergenerational Conversation Using Visual Cues: Effects of a Historical Timeline [M.S.]. Ann Arbor: University of South Florida; 2020. |
|  | 11. LoBuono DL, Leedahl SN, Maiocco E. Older adults learning technology in an intergenerational program: Qualitative analysis of areas of technology requested for assistance. Gerontechnology. 2019;18(2):97-107. |
|  | 12. MacDonald M. Intergenerational interactions occurring within a shared reading program. Journal of Intergenerational Relationships. 2005;3(4):45-61. |
|  | 13. Strand KA, Francis SL, Margrett JA, Franke WD, Peterson MJ. Intergenerational exergaming physical activity program increases flexibility and strength in older adults. FASEB Journal. 2012;26(Meeting Abstracts). |
| Duplicate Studies | 1. Aday RH, Rice C, Evans E. Intergenerational Partners Project: a model linking elementary students with senior center volunteers. The Gerontologist. 1991;31(2):263-6. |
|  | 2. Allred GB. THE EFFECTS OF INTERGENERATIONAL REMOTIVATION THERAPY ON THE LIFE SATISFACTION OF INSTITUTIONALIZED ELDERLY (OLDER ADULTS, NURSING HOMES) [Educat.D.]. Ann Arbor: Oklahoma State University; 1985. |
|  | 3. Ann-Kristin B. Intergenerational Learning in Stockholm County in Sweden: a practical example of elderly men working in compulsory schools as a benefit for children. Journal of Intergenerational Relationships. 2003;1(4):7-24. |
|  | 4. Arrington NM. The Effects of Participating in a Service-Learning Experience on the Development of Self-Efficacy for Self-Regulated Learning of Third Graders in an Urban Elementary School in Southeastern United States: ProQuest LLC; 2010. |
|  | 5. Berry CC. Impact of a task oriented intergenerational service learning program on two populations: UMI Dissertation Services, ProQuest Information and Learning, Ann Arbor, MI; 2003. |
|  | 6. Brant JL. The impact of consistent interaction with the elderly on social and emotional competency skills of 4 year olds: A mixed methods study. Dissertation Abstracts International Section A: Humanities and Social Sciences. 2019;80(11-A(E)):No-Specified. |
|  | 7. Cohen-Mansfield J, Jensen B. Intergenerational Programs in Schools. Journal of applied gerontology: the official journal of the Southern Gerontological Society. 2017;36(3):254-76. |
|  | 8. Freedman M. Partners in growth: elder mentors and at-risk youth: Public/Private Ventures, Philadelphia, PA; 1988. |
|  | 9. Lisbeth D, Dominic A, J SH. Making intergenerational connections: an evidence review. What are they, why do they matter and how to make more of them. 2017:74. |
|  | 10. McKee LL, Heydon RM. Orchestrating literacies: Print literacy learning opportunities within multimodal intergenerational ensembles. Journal of Early Childhood Literacy. 2015;15(2):227-55. |
|  | 11. Rosebrook V. Intergenerational Personal/Social Skills Development Study. Childhood Education. 2007;83(3):162. |
|  | 12. Sherman A. An exploration of intergenerational relations through dance between profoundly deaf individuals who are “total communicators” [Ph.D.]. Ann Arbor: New York University; 1993. |
|  | 13. Sikora SA. The University of Arizona College of Medicine Optimal Aging Program: stepping in the shadows of successful aging. Gerontology & geriatrics education. 2006;27(2):59-68. |
|  | 14. Sowle AJ. Intergenerational physical activity programming for rural-residing older adults [M.S.]. Ann Arbor: Iowa State University; 2015. |
|  | 15. Voglino G, Gualano MR, Bert F, Camussi E, Thomas R, Siliquini R. The impact of intergenerational programs on children and elder adults. European Journal of Public Health. 2017;27:410-. |
|  | 16. Wagner L. GENERATION TO GENERATION: EFFECTS OF CONNECTING OLDER AND YOUNGER ADULTS IN A COLLEGE SETTING. Gerontologist. 2016;56:9-. |
