# Supplementary material for: Non‐familial intergenerational interventions and their impact on social and mental wellbeing of both younger and older people—A mapping review and evidence and gap map
Source: Campbell Syst Rev. 2023 Feb 16;19(1):e1306. doi: 10.1002/cl2.1306 (PMC9934919; doi:10.1002/cl2.1306)
Supplement: Supplementary file 2 — Supplementary Information [file CL2-19-e1306-s001.docx]

**Abbreviations and acronyms**

[This will be a heading at the same level as Abstract at publication stage.]

EGM - Evidence Gap Map
